# Behavioral and Cellular Tagging in Young and in Early Cognitive Aging

**DOI:** 10.3389/fnagi.2022.809879

**Published:** 2022-02-24

**Authors:** Alexandra Gros, Amos W. H. Lim, Victoria Hohendorf, Nicole White, Michael Eckert, Thomas John McHugh, Szu-Han Wang

**Affiliations:** ^1^Centre for Clinical Brain Sciences, The University of Edinburgh, Edinburgh, United Kingdom; ^2^Canadian Centre for Behavioural Neuroscience, University of Lethbridge, Lethbridge, AB, Canada; ^3^Laboratory for Circuit and Behavioral Physiology, RIKEN Center for Brain Science, Saitama, Japan

**Keywords:** cognitive aging, memory consolidation, proximal and distal hippocampus, immediate early genes, *in situ* hybridization, behavioral tagging, trisynaptic circuit

## Abstract

The ability to maintain relevant information on a daily basis is negatively impacted by aging. However, the neuronal mechanism manifesting memory persistence in young animals and memory decline in early aging is not fully understood. A novel event, when introduced around encoding of an everyday memory task, can facilitate memory persistence in young age but not in early aging. Here, we investigated in male rats how sub-regions of the hippocampus are involved in memory representation in behavioral tagging and how early aging affects such representation by combining behavioral training in appetitive delayed-matching-to-place tasks with the “cellular compartment analysis of temporal activity by fluorescence *in situ* hybridization” technique. We show that neuronal assemblies activated by memory encoding were also partially activated by novelty, particularly in the distal CA1 and proximal CA3 subregions in young male rats. In early aging, both encoding- and novelty-triggered neuronal populations were significantly reduced with a more profound effect in encoding neurons. Thus, memory persistence through novelty facilitation engages overlapping hippocampal assemblies as a key cellular signature, and cognitive aging is associated with underlying reduction in neuronal activation.

## Introduction

Memory decline is commonly studied at a later stage of aging (>20 months old in rodents, Kausler, 1994; Bettio et al., 2017), while how memory is affected at midlife remains relatively understudied. Characterizing early signs of cognitive changes and correlating these changes with underlying cellular mechanisms in the brain would enable better understanding of the early stages of cognitive aging.

Memory persistence can be improved by introducing novelty around the time of memory encoding, a process called behavioral tagging (Vishnoi et al., 2016; Nomoto and Inokuchi, 2018). The event to be remembered (i.e., memory of interest) is hypothesized to induce molecular changes in cells and synapses in the brain (Moncada et al., 2015; Nomoto and Inokuchi, 2018). Environmental novelty (i.e., a memory-modulating event), when introduced around memory encoding, can modulate memory persistence (Moncada and Viola, 2007; Ballarini et al., 2009; Wang et al., 2010; Gros and Wang, 2018), upregulate the expression of immediate early genes (IEGs) (Guzowski and Worley, 2001), and induce the expression of plasticity-related proteins (Moncada and Viola, 2007; Ballarini et al., 2009; Almaguer-Melian et al., 2012). Behavioral tagging provides a robust method to sustain everyday memories (Wang et al., 2010; Nonaka et al., 2017). This strategy is, however, ineffective to maintain memory persistence in early aging (Gros and Wang, 2018), and the cellular correlates of behavioral tagging in cognitive aging need to be investigated.

Memories are associated with the activation of specific neuronal assemblies in the brain (Reijmers et al., 2007; Liu et al., 2012). Learning or novelty leads to IEG expression in neurons, which can be used to image neuronal activation following behavioral experiences (Kubik et al., 2007). The synaptic tagging and capture hypothesis posits that induction of potentiation *via* a weak stimulation triggers setting of tags at activated synapses. These tags then capture plasticity-related proteins that are triggered by strong stimulation. This process contributes to long-term plasticity changes (Frey and Morris, 1998). Applying this principle at the behavioral level, the prediction is that two neuronal assemblies that are activated by the encoding of an everyday memory and by the memory-modulating event need to be integrated into an overlapping neuronal assembly for behavioral tagging to occur. This leads to the hypothesis that during behavioral tagging there will be a significant increase in overlap of neuronal assemblies representing encoding and memory modulation. A mouse study revealed that weak object recognition followed by novelty engages overlapping hippocampal neuronal assemblies, a phenomenon called cellular tagging (Nomoto et al., 2016). Here, we aim to demonstrate whether this cellular property is changed during the aging process that coincides with the decline in everyday memory and behavioral tagging. We also characterized the cellular signatures among subregions of the hippocampus. Anatomical and functional data suggest that different kinds of information related to an item’s features and spatial characteristics are encoded in separate pathways in the CA1, CA3, and dentate gyrus (DG). Particularly, the distal CA1-proximal CA3 and the proximal CA1-distal CA3 circuits are differentially involved in encoding spatial and non-spatial information (Burke et al., 2011; Sauvage et al., 2013; Flasbeck et al., 2018; Hoang et al., 2018).

We combined behavioral training in an appetitive delayed-matching-to-place (ADMP) task with the “cellular compartment analysis of temporal activity by fluorescence *in situ* hybridization” (catFISH) technique to label neuronal assemblies involved in this process (Guzowski et al., 2005). CatFISH uses the temporal and subcellular distribution of two IEGs, *Arc* and *Homer1a (H1a)*, to infer the activation history of neuronal assemblies during two epochs of behavior. We asked if an effective modulating event (novelty), as opposed to an ineffective one (familiarity) that occurs after encoding, would trigger more neurons co-expressing *Arc* and *H1a*. Critically, we characterized this cellular signature change with aging and the neuronal activation patterns across CA1, CA3, and DG in young animals. We showed that behavioral tagging involves a shared hippocampal neuronal assembly in distal CA1 and proximal CA3, and early aging leads to significant reduction of activated neuronal populations that are associated with memory decline.

## Materials and Methods

### Experimental Model and Subject Details

Adult male Lister Hooded rats (Charles River, 10 to 12 weeks on arrival, *n* = 61) were group housed (4 per cage) in a temperature- and humidity-controlled colony room. The sample size calculation was based on previously published work using the ADMP task and catFISH technique. The room was under a 12-h light/dark cycle (light onset, 7:00 AM), and behavioral training and testing were conducted during the light phase (between 9:00 AM and 5:00 PM). The rats were handled for 5 days during which food and water were available *ad libitum*. Access to food was restricted per cage at 20–25 grams on average per rat per day to maintain each rat’s body weight at around 90–95% of free-feeding weight during behavioral procedures. All experiments were approved by the institutional veterinary service and performed in accordance with the United Kingdom Home Office regulations of animal experimentation [Animals (Scientific Procedures) Act 1986, Amendment Regulations 2012].

### Experimental Design

**FIGURE 1:**
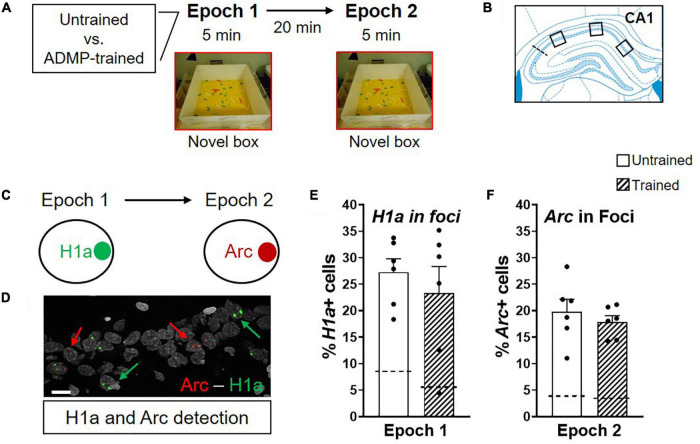
ADMP training does not change the proportion of *Arc*+ and *H1a* + neurons after novel box exploration. **(A)** Experimental design. ADMP-trained or untrained young rats explored a novel box during epoch 1 and/or epoch 2, separated by 20 min. **(B)** Images were collected in 3 areas of the CA1. **(C)** *H1a* and *Arc* expression in nuclei was used to quantify neuronal activation after novel box exploration during epoch 1 or epoch 2, respectively. **(D)** An example of CA1 cells showing *H1a* + (the green arrow) or *Arc*+ (the red arrow). A scale bar: 10 μm. **(E)** ADMP training did not change the percentage of *H1a* + neurons activated by novelty exploration during epoch. **(F)** ADMP training did not change the percentage of *Arc*+ neurons activated by novelty exploration during epoch 2. All data are presented as mean ± SEM. The dashed line represents the basal level of activation without novel box exploration.

**FIGURE 2:**
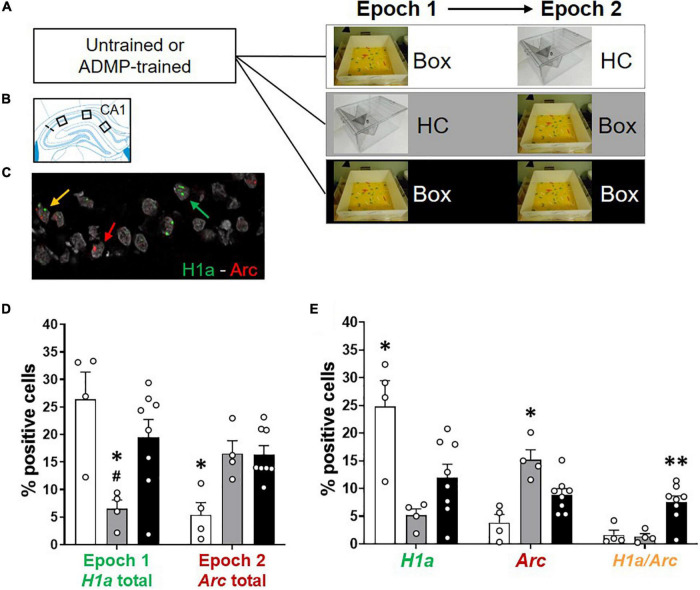
Re-exposure to the same novel environment increases the proportion of overlapping CA1 neurons. **(A)** Experimental design. ADMP-trained and untrained young rats received one or two behavioral events (5-min epoch 1, 20-min delay, and 5-min epoch 2) in three groups: (1) exploration in a novel box in epoch 1 and a home cage in epoch 2 (Box – HC), (2) a home cage in epoch 1 and exploration in a novel box in epoch 2 (HC – Box), or (3) exploration in a novel box in both epochs (Box – Box). **(B)** Three areas of image collection in the CA1 subregion of the hippocampus. **(C)** An example of CA1 neurons expressing *H1a* (the green arrow), *Arc* (the red arrow), or both *H1a/Arc* (the yellow arrow). A scale bar: 10 μm. **(D,E)** Automatic detection of overlapping CA1 neurons after re-exposure to the same novel box. **(D)** Left. Percentage of neurons expressing *H1a* (with or without co-expressing *Arc*) showed increase by the novel box exploration in epoch 1. #, Box + HC vs. HC + Box, *p* = 0.062. Right. Percentage of neurons expressing *Arc* (with or without co-expressing *H1a*) showed increase by the novel box exploration in epoch 2. **(E)** Left. Percentage of neurons expressing *H1a* only (without co-expressing *Arc*) showed increase by novelty during epoch 1. Middle. Percentage of neurons expressing *Arc* only (without co-expressing *H1a*) showed increase by novelty during epoch 2. Right. Percentage of neurons co-expressing *H1a/Arc* showed an increase when novelty was introduced two times. All data are presented as mean ± SEM. *p < 0.05 and **p < 0.01.

Three cohorts of rats were used in three experiments. Experiment I (Figures 1, 2) aimed at (1) validating the imaging technique, (2) replicating previous findings in showing preferential activation of *H1a* and *Arc* for two temporally separate events, and (3) examining if prior training of the ADMP task would not change the profile of *Arc* and/or *H1a* expression (Figure 1). For this, young rats (*n* = 8) were trained in the ADMP task at the age of 3 to 4 months, and the other group (*n* = 8) was not trained. For the trained group, the young rats received 12 training sessions followed by various probe tests with interleaving training sessions to evaluate their memory persistence. The *in vivo* experiment ended with the introduction of two behavioral epochs on the final critical day: epoch 1 referred to 25–30 min before the brain extraction, while epoch 2 referred to 0–5 min before the brain extraction. The trained and untrained rats were randomly assigned to one of the three following groups (Figure 2A): the rats in group 1 were placed in a novel box during epoch 1 and then remained in their home cage during epoch 2 (Box – HC, *n* = 4, 2 were ADMP trained); the rats in group 2 stayed in their home cage during epoch 1 and then were placed in a novel box during epoch 2 (HC-Box, *n* = 4, 2 were ADMP trained); the rats in group 3 were placed in a novel box during epoch 1, returned to their home cage, and placed in the same box during epoch 2 (Box – Box, *n* = 8, 4 were ADMP trained).

Experiments II-III (Figures 4–8) examined the activation of hippocampal neuronal assemblies involved in behavioral tagging in young (Experiment II) and in middle-aged animals (Experiment III). For this, 25 young rats were trained and tested at the age of 3 to 5 months, 16 middle-aged rats were trained and tested at the age of 8.5 to 12 months old, and 4 untrained middle-aged rats were included in the home cage control group. Great care was taken to ensure the same experimenters conducted the studies in a similar environment with similar procedures using similar genetic and early-life background of animals. The consistency in animals’ behavioral performance in tissue preparation, in catFISH, and in cellular image acquisition and quantification was monitored and verified across cohorts in the lab.

Appetitive delayed-matching-to-place (ADMP) training was composed of 12 sessions of encoding-retrieval trials and followed by various probe tests, with interleaving training sessions, to evaluate their memory persistence. All the rats were randomly divided into the following groups on the final critical day: the rats in group 1 remained in the home cage (HC + HC, young *n* = 5, middle-aged *n* = 4) for both epochs; the rats in group 2 received an encoding trial (with 3 pellets) in the ADMP task during epoch 1 (Enc + HC, young *n* = 3, middle-aged *n* = 5); the rats in group 3 were exposed to a novel box during the epoch 2 (HC + Nov, young *n* = 3); the rats in groups 4 and 5 received an encoding trial (with 3 pellets) in the ADMP task during epoch 1, returned to the home cage for 20 min, and then were placed in a novel or familiar box (Enc + Nov: young, *n* = 7; middle-aged, *n* = 6; Enc + Fam: young, *n* = 7; middle-aged, *n* = 5). The encoding on the final day was composed of 3 pellets to ensure that sufficient encoding-trigger H1a expression beyond baseline expression in the hippocampus would be observed. Pevzner et al. (2012) showed that CA1 can fully express IEGs after 30 s of experience, but it was an *Arc* effect driven by novelty (Pevzner et al., 2012). We do not know if 30-s encoding in a familiar environment would lead to a significant increase of *H1a* mRNA expression beyond the expression level seen in the home cage. Familiar environment is shown to not improve memory retention (Ballarini et al., 2009; Wang et al., 2010). The brains were collected immediately after epoch 2. The detection of *H1a* and *Arc* mRNA in foci distinguished neuronal populations activated by ADMP encoding (memory of interest) and novelty (a memory-modulating event), respectively.

For effective comparison, data may be grouped based on whether animals underwent a procedure or remained in the HC in each epoch. For epoch 1, no encoding (No Enc) referred to all animals remained in the HC in epoch 1 regardless of whether they were placed in a box in epoch 2. Encoding (Enc) referred to all animals that received an encoding trial regardless of whether they were placed in a box in epoch 2. For epoch 2, “No Box” referred to all animals that remained in the HC. “Box” referred to all the animals that were placed in a novel or familiar box. Good temporal differentiation between *H1a* and *Arc* mRNA expression in foci (Guzowski et al., 2005) enables this type of epoch-based comparisons.

### Behavioral Procedure

#### Apparatus for the Appetitive Delayed-Matching-to-Place Task

**FIGURE 3:**
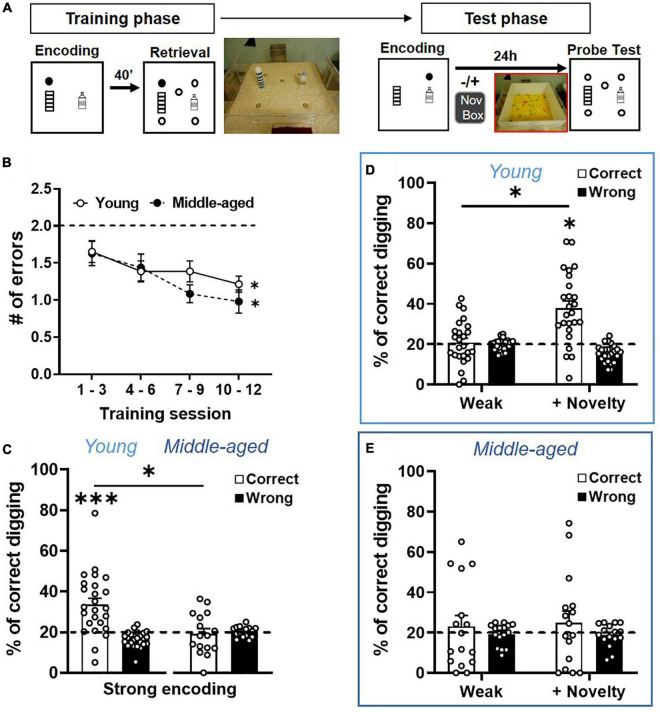
Long-term memory persistence and modulated memory persistence are impaired in aging**. (A)** Behavioral procedures of ADMP training and testing. Rats were trained to find hidden rewards in a sand well (the filled circle) inside the arena. After a delay, the rats had access to the arena with five sand wells, but only the matching location, which was rewarded in the encoding phase, contained more rewards. There were 12 encoding-retrieval training sessions. Memory retention was tested after weak (1 pellet) or strong (3 pellets) encoding, at 24-h delay, in a non-rewarded probe trial (open circles). Exploration of a novel environment (box) could be introduced 30 min after encoding. **(B)** The number of errors decreased across the 4 blocks of training sessions in the young and middle-aged rats. The dashed line indicates the chance level. **(C)** Long-term memory retention after strong encoding showed that the percentage of correct digging was significantly above chance (the dashed line) in the young rats, but not in the middle-aged rats. **(D)** Long-term memory retention after weak encoding showed performance near chance (the dashed line), while novelty significantly improved the memory. **(E)** Long-term memory retention after weak encoding showed performance near chance, and novelty did not improve the memory. All data are presented as mean ± SEM. *p < 0.05 and ^***^p < 0.005.

Appetitive delayed-matching-to-place (ADMP) training was conducted in an event arena (135 cm × 135 cm × 40 cm, made of clear Plexiglas walls and white Plexiglas floor, Figure 3A) lined with ∼2-cm sawdust and contained 2 intra-maze landmarks. The arena was placed in a rectangular experimental room with extra-maze visual cues. Further details can be seen in Gros and Wang (2018). Four start boxes (30 cm × 25 cm × 30 cm) were placed in the center of each wall, covered with red filter paper that darkened the box, and equipped with automated doors under the control of the experimenter. Chocolate-flavored food pellets (0.5 g per rewarded pellet, Supreme Mini Treats™, ref: F05472, Bio-Serv) were used as rewards in this task. Plexiglas sand wells (6-cm diameter, 4-cm depth) could be inserted into the floor of the arena at different locations. To mask olfactory cues emanating from the reward, the sand wells were filled with bird sand mixed with 5% of ground pellets. In addition, at the bottom of every sand well, 4 g of food pellets were kept out of reach of the animals by a metal mesh divider.

#### Box for Exploration

A square (100 cm × 100 cm) Plexiglas box with white walls (40 cm) was used (Figure 3A). To introduce novelty (Diamond, 2010), we placed green, gold, and gray aquarium pebbles (varied from 0.3 cm to 1 cm in diameter) on the white Plexiglas floor. On the final critical day before tissue collection, 1 cm × 2 cm × 3 cm ceramic bricks were placed on the floor to introduce novelty, which has been shown to improve memory persistence (Wang et al., 2010).

#### Appetitive Delayed-Matching-to-Place Training Procedures

The rats were first habituated to the event arena and procedures and received training with the ADMP task. After establishing the training, their memory persistence was examined in various probe tests with different encoding strengths, time delay after encoding, and with or without novelty after encoding.

##### Habituation

Phase 1: The rats were handled every day for 5 days to habituate them to the experimenter and reduce their stress level. They were weighed daily to establish the baseline of weight gain under normal feeding. Phase 2: Sand wells with chocolate-flavored pellets were placed in the home cages. The rats had two session to dig through the sand and eat pellets, and food restriction began and remained for the rest of the procedures. Phase 3: The rats explored a quarter of the event arena (divided by removable walls) with a sand well containing four pellets (one pellet on top and three pellets in the middle of the sand well). They had 4 sessions for each quadrant to dig through the sand and eat pellets. They then explored half of the event arena to dig the sand well for pellets. Finally, they explored the whole event arena with a rewarded sand well placed at the center of the arena. They would typically find the pellet and carry it to the start box to eat. Each habituation session stopped when they found and ate 3 pellets and capped at 15 min.

##### Training

The rats were trained in the event arena for 12 sessions. A daily training session consisted of a memory-encoding trial followed by a retrieval trial about 40 min later. During the encoding trial, one rewarded sand well was placed in the arena at a particular location and constituted the opportunity for each rat to encode where the food was available on that day. The rewarded location (e.g., far from or near the start box) on a given day was counter-balanced across all the rats, and the same location was not repeated for consecutive trials to avoid bias toward certain part of the arena. The rewarded location for a given rat was also counter-balanced across training sessions to avoid the development of egocentric bias. The rats were given a pellet in the start box to accustom the animals to eat at the start box. The door was then opened, remotely controlled by the experimenter. The rats explored the arena, found the sand well location, dug for the hidden reward, and then carried the pellet back to the start box to eat. They repeated these procedures until they collected 3 pellets. At the retrieval trial, 5 different sand well locations were present, but only the same sand well location that matched the encoding trial would contain rewards. If the rewarded location during the encoding trial was remembered, the animal would return to the matching location to find more rewards. The trial ended after the rats had found and eaten 3 pellets. The rewarded sand well location and the start box location (north, east, south, and west) would change across sessions to encourage the animal to encode a new location in different sessions.

##### Probe Tests

After 12 training sessions, the rats received various encoding-probe sessions that were interleaved with regular encoding-retrieval training sessions. A typical condition session consisted of a rewarded encoding trial, followed by a probe trial with five non-rewarded sand wells (Wang et al., 2010; Salvetti et al., 2014). The probe trial was 60-s long with one of the five sand wells placed at the matching location to the encoding trial. After 60 s, the experimenter placed 1 pellet at a surface and 2 pellets at the bottom of the matching sand well so the rats could find and eat the pellets. This was to avoid weakening of using the matching search strategy after a probe test. To evaluate if novelty after encoding facilitated memory persistence, the rats received the encoding trial, returned to their home cage, and 30–40 min later were placed in a novel box for 5 min of exploration. Counterbalancing between paired conditions (e.g., with and without novelty) was carried out carefully.

##### Familiarization to the Box

For the encoding-familiarity group, the rats were habituated to the box for 20 min each day for 3 consecutive days prior to the final critical day. The same Plexiglas box with small bricks on the floor was used to match the group that received encoding novelty without familiarization. Hence, the differential change in cellular signature between these 2 groups would most likely be explained by the level of novelty.

### Behavioral Analysis

During the training, the accuracy of memory was measured by the number(s) of non-matching (*i.e.*, wrong) sand wells the rats had dug in before they dug in the correct sand well. The efficiency of retrieval was measured by the latency (in seconds) to find pellets in the rewarded sand well. The learning curve was typically characterized by a reduction of errors or latency across the training sessions. For assessing memory at probe tests, the time that the rats spent digging (contact the sand well with forepaws that led to displacement of sand) in different sand wells was recorded for the first 60 s of the trial. The percentage of time spent digging at the correct location over the total digging time constituted the correct digging percentage. The average of percentage of time digging at the non-matching locations over the total digging time constituted the wrong digging percentage. A custom-built LabView timer was used to record the digging time and latency. All measurements were done by the experimenters who were blind to the probe conditions or group identity.

### Tissue Preparation

On the final critical day, as described in the “experimental design,” an experienced technician rapidly performed a neck dislocation and decapitated the animals with a rodent guillotine immediately after epoch 2. The brains were swiftly extracted and flash frozen in isopentane at around −45°C in dry ice. All the brains were stored at −80°C until further processing. Coronal sections (20 μm) were collected using a cryostat and mounted on slides (Superfrost plus slides, VWR, United Kingdom). The slides were air dried and stored at −80°C.

### Fluorescent *in situ* Hybridization (Compartment Analysis of Temporal Activity by Fluorescence *in situ* Hybridization)

Brain sections underwent single- or double-label fluorescence *in situ* hybridization (FISH) to detect neurons expressing *Homer1a* and/or *Arc* as described before (Chawla et al., 2004; Sauvage et al., 2019). A commercial T3 polymerase transcription kit (MaxiScript, AM1316, Thermo Fisher Scientific, United Kingdom) and premixed RNA labeling nucleotide mixes containing either digoxigenin- or fluorescein-labeled UTP (11277073910 Roche and 11685619910 Roche, Merck, United Kingdom) were used to generate *Arc* and *H1a* antisense riboprobes, respectively (plasmids courtesy of J. Guzowski). Riboprobes were purified on MicroSpin™ G-50 Columns (GE Healthcare, United Kingdom). The yield and the integrity of riboprobes were confirmed by gel electrophoresis. The quantity of riboprobes was determined by NanoDrop (NanoDrop One, Thermo Fisher Scientific, United Kingdom). The *Arc* intron-enriched riboprobe detected *Arc* transcription foci, but not cytosolic/dendritic mRNA efficiently (Guzowski et al., 2006), and the *H1a* antisense riboprobe detected mRNA in foci reliably (Kubik et al., 2012).

#### Single Arc Fluorescence *in situ* Hybridization

Brain sections were fixed with cold 4% buffered paraformaldehyde, treated with 0.5% acetic anhydride/1.5% triethanolamine, methanol:acetone at −20°C, and equilibrated in 2 × Saline Sodium Citrate (SSC). Slides were incubated in a 1 × prehybridization buffer (Merck) for 30 min at room temperature. *Arc* antisense riboprobes were diluted to 0.75 ng/μl in a 1 × hybridization buffer (Merck), denatured by heat, chilled on ice, and subsequently added to each slide. Hybridization was carried out at 56°C overnight. A 14 h later, the slides were washed at 37°C in 2 × SSC with RNase A (0.01 mg/ml) before reaching in a final stringency of 0.5 × SSC at 56°C during 30 min. H_2_O_2_ (2%) in 1 × SSC was employed to suppress endogenous peroxidase activity. The slides were blocked with a.1-M Tris-HCl/0.15-M NaCl blocking buffer in 5% sheep serum for 1 h. *Arc* riboprobes were then detected by incubating with anti-digoxigenin-POD HRP (1/400, Roche) at room temperature for 2 h. The slides were washed in Tris-buffered saline with 0.05% Tween-20 (TBS-T), and the conjugate was detected with cyanine-5 substrates (Perkin Elmer TSA Plus). After final TBS-T and TBS washes, the slides were mounted in a Vectashield mounting medium with 4′, 6′-diamidino-2-phenylindole (DAPI, Vector Labs), and coverslips were applied.

#### Double H1a/Arc Fluorescence *in situ* Hybridization

The sections were processed in the same way except the following. Hybridization was done with both *Arc* and *H1a* antisense riboprobes (0.75 ng/μl each). After RNase A and H_2_O_2_ treatment, the riboprobe detection was done sequentially. *H1a* riboprobes were first detected by incubation with anti-fluorescein horseradish peroxidase (HRP)-antibody conjugate (Perkin Elmer TSA Plus) for 2 h at room temperature. The slides were washed in TBS-T, and the detection was done with a cyanine-3 substrate kit (Perkin Elmer TSA Plus). H_2_O_2_ in TBS (2%) was used to quench residual HRP activity. *Arc* riboprobes were detected by incubating in anti-digoxigenin-POD HRP (Roche) at 4°C overnight. A 15 h later, the slides were washed in TBS-T, and the conjugate was detected with cyanine-5 substrates (Perkin Elmer TSA Plus).

### Image Acquisition and Analysis

The images were acquired using a Zeiss LSM 710 confocal microscope with a krypton-argon laser. The photomultiplier tube (PMT) gain, pinhole sizes, and contrast settings were kept constant for all image stacks across brain and cohorts. The settings were optimized for detecting only strong intranuclear signals and for minimizing background by adjusting the laser power settings and PMT gain. Two slides per brain were selected around Bregma -3.5 mm to represent dorsal hippocampus and to keep anatomical consistency. Confocal z-stacks composed of 1-μm thick optical sections were collected in the dorsal CA1, CA3, and DG by using a 40 × oil objective. The CA1 and CA3 were localized on each cut using the Paxinos & Watson rat brain atlas (5th edition). Three stacks of CA1 images were sampled equally across the proximal/distal axis: the first one before the CA1 curve close to the fascia cinereum, one in middle just after the CA1 curve, and the last one toward the end of CA1 (Figure 1B). Two stacks in CA3 were sampled equally across the proximal/distal axis: lateral side of CA3 and near the DG (Figure 8A). Five z-stacks in DG (2 from the suprapyramidal/outer blade, 2 from the infrapyramidal/inner blade, and 1 from the apex) were collected (Figure 8F).

Images were analyzed using Fiji/ImageJ open-source software (NIH, Bethesda, MD) by experimenters who were blind to the experimental conditions. The first and last 20% of images of each z-stack were excluded from the analysis to avoid bias. The total number of DAPI-positive cells was counted per stack by the experimenters who were blind to the animal’s group identity. Non-neuronal cells were identified based on their extremely and uniformly bright nuclei and their small soma size (∼5 μm in diameter), likely to be glial cells, and were not counted. Comparable numbers of cells per z-stack were collected for all the groups (Table 1; one-way ANOVA: Experiment I, *Arc* only, F_2,13_ = 0.15; *Arc/H1a*, F_2,13_ = 0.94, *p* = 0.42; Experiment II, *Arc/H1a*, CA1, F_4,18_ = 0.8, *p* = 0.54; CA3, F_4,18_ = 1.7, *p* = 0.19; DG, F_4,18_ = 0.25, *p* = 0.9; Experiment III, *Arc/H1a*, CA1, F_3,16_ = 0.38, *p* = 0.77). The number of cells analyzed was similar to previous studies (Guzowski et al., 1999, 2006).

**TABLE 1:** Mean of the number of cells per z-stack that was counted in sub-regions of the hippocampus.

		Group	Hippocampus		
			CA1	CA3	DG
Experiment I	*Arc* only	Box + HC	48.8 ± 2.4		
		HC + Box	54.8 ± 5.9	NA	NA
		Box + Box	65.2 ± 4.4		
	*Arc/H1a*	Box + HC	45.5 ± 4.7		
		HC + Box	51.7 ± 4.1	NA	NA
		Box + Box	49.4 ± 1.2		
Experiment II	*Arc/H1a*	HC + HC	51.3 ± 2.4	34.4 ± 1.6	195.6 ± 9.7
		Enc + HC	46 ± 6.3	27.4 ± 2.2	205.7 ± 22.4
		HC + Nov	45.1 ± 4.3	29.8 ± 1.8	214 ± 25.4
		Enc + Nov	45.8 ± 1.4	29.4 ± 1.9	215.5 ± 22
		Enc + Fam	45.1 ± 2	30.3 ± 1.3	200.4 ± 7.9
Experiment III	*Arc/H1a*	HC + HC	49,8 ± 3,5		
		Enc + HC	54,6 ± 5.9		
		Enc + Nov	54,8 ± 4,8	NA	NA
		Enc + Fam	57,6 ± 4,6		

#### Single Arc Fluorescence *in situ* Hybridization

*Arc* foci and cytoplasm signals were first detected manually by an experimenter blind to the animal’s group identity. Four types of cells were classified: with or without *Arc* signal in foci or in cytoplasm. A cell was counted as *Arc*+ if the signal was present on two or more consecutive images per z-stack. A cell was counted as expressing *Arc* mRNA in the cytoplasm if the signal was distributed surrounding the nucleus. The results were expressed as a percentage of the total cells per z-stack.

#### Double H1a/Arc Fluorescence *in situ* Hybridization

*Arc* and *H1a* foci signals were detected using a purpose-built script in Fiji/ImageJ for automatic detection. This script enables detection of *Arc* (red signal) and *H1a* (the green signal) signals within the nucleus (the blue signal from DAPI) using co-localization between the red or the green signal and the blue signal. The red and green signals are counted if the number of pixels was larger than 15, intensity higher than 40, and present on 2 or more z-stacks. The reliability of the automatic detection was verified through manual detection (Figure 2).

Four types of cells were classified: with or without *Arc* or *H1a* in foci. The results were expressed as a percentage of the total cells per stack. In addition to counting cell numbers, we explored if there was a change of *Arc* or *H1a* signal size in CA1. “Blob volume” was generated from the *Arc* or *H1a* signal in nuclei by using Color Pixel Counter plug-in in Fiji/ImageJ (pixel intensity fixed at 40). The number of pixels that compounded one blob was measured on continuous z-stack and added together to obtain the volume of the blob.

### Statistical Analysis

#### Behavior Analysis

For behavioral data, parametric tests were used, as the data (*n* = 33 from the trained animals) conformed to a normal distribution (Shapiro-Wilk test). Training data were presented as 4 blocks of 3 training sessions. Dunnett’s tests were used to compare block 1 with subsequent blocks. The number of errors across the blocks was analyzed using repeated-measures one-way ANOVA. One-sample *t*-tests were used to compare each block with the chance level, which was 2. One-sample *t*-tests were used to compare the percentage of digging in the correct sand well with the chance level, which was 20%. Paired *t*-tests were used to compare the percentage of correct digging between 2 different conditions.

#### Compartment Analysis of Temporal Activity by Fluorescence *in situ* Hybridization Analysis

The normality of the data is checked using Shapiro-Wilk test. If the data followed a normal distribution, then parametric tests were used: unpaired *t*-test to compare two groups or one- or two-way ANOVA to compare multiple groups followed by *post hoc* Tukey multiple-comparison tests. If the data did not follow a normal distribution, then non-parametric tests were used: Mann-Whitney test to compare two groups or Kruskal-Wallis test to compare multiple groups followed by *post hoc* Dunn multiple-comparison test.

#### Blob Volume

To explore the trend of blob size changes, individual blobs were initially used as the analysis units to enhance the statistical power, followed by averaging individual blobs per animal and using animals as units for analyses. Similarly, if the data were normally distributed, parametric tests were used; otherwise, non-parametric tests were applied as described above.

Data (animals as the statistical units) were presented as mean ± SEM. All statistical analysis was done using GraphPad Prism 9. All statistical significance was set at *p* < 0.05.

## Results

### Prior Training Does Not Change the Proportion of Novelty-Activated Neurons

We first examined whether prior training in the ADMP task would or would not change the level of IEG expression triggered by the exploration of a novel box, a procedure commonly used in catFISH studies (Guzowski et al., 1999; Vazdarjanova et al., 2002). Neuronal activation leads to rapid *Arc* mRNA expression in the nucleus of activated neurons, whereas after a 20–30 min delay, *Arc* mRNA is translocated to the cytoplasm and *H1a* mRNA expression becomes apparent in the nucleus (Vazdarjanova et al., 2002; Guzowski et al., 2005). IEGs are expressed predominantly, although not exclusively, in excitatory neurons, but not in glial cells (Vazdarjanova et al., 2006). Therefore, cells expressing IEGs in this context were considered neurons.

The young adult rats were randomly assigned to receive ADMP training or not. On the final critical day, the animals were randomly assigned to one of the following groups: exploring a novel box in epoch 1 [Box + home cage (HC) group, expecting to trigger *H1a* expression in foci or *Arc* expression in cytoplasm], in epoch 2 (HC + Box group, expecting to trigger *Arc* expression in foci), or in both epochs (Box + Box group) (Figures 1A, 2A). We first asked if previous training with the ADMP task would change the proportion of CA1 cells expressing IEGs after exploration in a novel box in both epochs. Results showed that novelty activated a similar proportion of *Arc*+ or *H1a*+ neurons in CA1 in the ADMP-trained or untrained rats (Figures 1C–F, trained vs. untrained, *t*_10_ = 0.7, *p* = 0.5; *t*_10_ = 0.73, *p* = 0.48; Supplementary Figures 1A–D, trained vs. untrained, unpaired *t*-tests, *t*_10_ = 1.34, *p* = 0.21; *t*_10_ =, *p* > 0.99), indicating that the level of IEG expression is not changed by prior ADMP training.

We next examined the profile of cellular IEG expression based on the behavioral experiences just before tissue collection (Figures 2A–C). We carefully used 3 methods: first, manual detection of Arc expression in cytoplasm or foci (Supplementary Figures 2A,B); second, manual detection of Arc and H1a expression in foci (Supplementary Figures 2C,D); third, ImageJ-scripted automatic detection of Arc and H1a expression (Figures 2D,E). The results show that all 3 methods led to a similar pattern of finding. The animals exploring a novel box in epoch 1 showed more total cytoplasmic *Arc*+ neurons or *H1a*+ neurons (i.e., *H1a* expression in foci or *Arc* expression in cytoplasm with and without *Arc* co-expression in foci) than animals which stayed in their home cage (Supplementary Figure 2A, left, one-way ANOVA *F*_2,13_ = 10.97, *p* = 0.002; Supplementary Figure 2C, left, one-way ANOVA *F*_2,13_ = 4.43, *p* = 0.034; Figure 2D, left, one-way ANOVA *F*_2,13_ = 5.88, *p* = 0.015; all Tukey tests are summarized in Table 2). The animals exploring a novel box in epoch 2 showed more total foci *Arc*+ neurons (*i.e.*, *Arc* expression in foci with and without *Arc* co-expression in cytoplasm or *H1a* co-expression in foci) than the animals which stayed in their home cage (Supplementary Figure 2A, right, one-way ANOVA *F*_2,13_ = 18.03, *p* = 0.0002; Supplementary Figure 2C, right, one-way ANOVA *F*_2,13_ = 7.55, *p* = 0.007; Figure 2D, right, one-way ANOVA *F*_2,13_ = 8.65, *p* = 0.004; all Tukey tests are summarized in Table 2).

**TABLE 2:** Comparisons between pairs of groups (Tukey tests) for Figure 2 and Supplementary Figure 2.

Tukey test – *p*-Value		Box + HC vs. HC + Box	Box + HC vs. Box + Box	HC + Box vs. Box + Box
Epoch 1	Supplementary Figure 1A	0.005 ***	0.98	0.002 ***
	Supplementary Figure 1C	0.054 #_1_	0.95	0.046 *
	Figure 2D	0.013 *	0.4	0.062 #
Epoch 2	Supplementary Figure 1A	0.0005 ***	0.0003 ***	0.92
	Supplementary Figure 1C	0.018 *	0.008 **	0.99
	Figure 2D	0.012 *	0.005 **	0.99
*Arc* in cyto	Supplementary Figure 1B	0.0001 ***	0.0003 ***	0.34
*H1a*	Supplementary Figure 1D	0.015 *	0.075 #_2_	0.36
	Figure 2E	0.004 **	0.023 *	0.27
*Arc* in foci	Supplementary Figure 1B	<0.0001 ***	<0.0001 ***	0.03
*Arc*	Supplementary Figure 1D	0.004 **	0.015 *	0.43
	Figure 2E	0.0007 ***	0.018 *	0.059
*Arc* in both	Supplementary Figure 1B	0.99	0.0006 ***	0.0007 ***
*H1a/Arc*	Supplementary Figure 1D	>0.99	0.005 ***	0.005 ***
	Figure 2E	0.99	0.008 **	0.006 **

^#^p < 0.08, *p < 0.05, **p < 0.01, and ***p ≤ 0.005.

Further quantification was performed by separating cells with only cytoplasmic *Arc* (or *H1a* in foci), only foci *Arc*, or both cytoplasmic and foci *Arc* (or both *H1a/Arc* in foci). The percentage of neurons containing only *Arc* in cytoplasm or *H1a* in foci was highest in the Box-HC group (Supplementary Figure 2B, left, one-way ANOVA *F*_2,13_ = 21.36, *p* < 0.0001; Supplementary Figure 2D, left, one-way ANOVA *F*_2,13_ = 5.67, *p* = 0.017; Figure 2E, left, one-way ANOVA *F*_2,13_ = 8.68, *p* = 0.004, all Tukey tests are summarized in Table 2). The percentage of neurons containing only foci *Arc* was highest in the HC-Box group (Supplementary Figure 1B, middle, one-way ANOVA *F*_2,13_ = 76.8, *p* < 0.0001; Supplementary Figure 2D, middle, one-way ANOVA *F*_2,13_ = 8.57, *p* = 0.004; Figure 2E, middle, one-way ANOVA *F*_2,13_ = 12.45, *p* = 0.001; all Tukey tests are summarized in Table 2).

When looking at the overlapping population, the percentage of *Arc*+ cyto/foci or *H1a/Arc*+ neurons was highest in the Box - Box group (Supplementary Figure 2B right, one-way ANOVA *F*_2,13_ = 18.72, *p* = 0.0001; Supplementary Figure 2D, right, one-way ANOVA *F*_2,13_ = 11.3, *p* = 0.001; Figure 2E, right, one-way ANOVA, *F*_2,13_ = 10.53, *p* = 0.002; all Tukey tests are summarized in Table 2). These percentages of *H1a*+, *Arc*+, and *H1a/Arc*+ neurons are consistent with previous work in which the animals voluntarily explored the environment (Nalloor et al., 2012; Kubík et al., 2014), but may be lower than other studies in which the animals were manually placed in every zone of the box (Vazdarjanova et al., 2002; Vazdarjanova and Guzowski, 2004; Marrone et al., 2008).

Comparison of the Fiji/ImageJ-scripted automatic detection vs. visual-manual detection of *H1a* and *Arc* signals in nuclei or the *Arc* signal in cytoplasm and nuclei revealed that both methods yielded similar patterns of activation, which supports the reliability of automatic detection. Together, we showed that repeated exploration of the same novel environment reactivated a significant proportion of CA1 neurons, as previously reported (Guzowski et al., 1999; Vazdarjanova and Guzowski, 2004). Moreover, prior ADMP training did not change the neuronal activation by novelty.

### Decline of Memory Persistence in Early Aging

After validating the cellular image approach, we characterized the behavioral performance and memory persistence in the ADMP task in the young and middle-aged rats. Two cohorts of the rats were trained to acquire the ADMP task, and the memory persistence was evaluated in subsequent probe tests (Figure 3A). Over 12 training sessions, the number of errors during retrieval gradually decreased and became significantly below chance, indicating that the rats learned the task (Figure 3B, young: a linear trend of reduction, one-way ANOVA *F*_3,24_ = 2.8, *p* = 0.038; Dunnett’s tests for comparison with block 1: block 2 *p* = 0.32, block 3 *p* = 0.044, block 4 *p* = 0.025; a middle-aged linear trend of reduction, one-way ANOVA *F*_3, 15 = _ 3.29, *p* = 0.029; Dunnett’s tests for comparison with block 1, block 2 *p* = 0.76, block 3 *p* = 0.067, block 4 *p* = 0.023). There was no significant difference between cohorts (two-way ANOVA, age effect *F*_1,39_ = 0.53, *p* = 0.47) or in the learning rate between the young and middle-aged rats (interaction effect *F*_3,39_ = 0.28, *p* = 0.84). Moreover, the latency to retrieve the 3 pellets showed a significant decrease across training sessions in both the young and middle-aged rats (data not shown; young: one-way ANOVA, training effect *F*_3,24_ = 26.20, *p* < 0.0001; Middle-aged: one-way ANOVA, training effect *F*_3,15_ = 40.31, *p* < 0.0001). There was no significant difference between cohorts (two-way ANOVA, age effect *F*_1,39_ = 0.97, *p* = 0.33) or in the learning rate between the young and middle-aged rats (interaction effect *F*_3,39_ = 2.076, *p* = 0.11). Together, the comparable learning rate indicates that early aging did not affect the animals’ ability to learn the ADMP task, and the comparable latency suggests that early aging did not affect the animals’ motivation or motor abilities at performing this task.

After strong encoding, good long-term memory (LTM, 24 h) was seen in the young rats (Figure 3C, one-sample *t*-test, *t*_24_ = 4.49, *p* = 0.0002), but not in the middle-aged rats (one-sample *t*-test, *t*_15_ = 0.3, *p* = 0.76). A significant aging effect was observed (unpaired *t*-test, *t*_39_ = 3.34, *p* = 0.002). Weak encoding led to poor LTM in both cohorts (Figure 3D, left, one-sample *t*-test, *t*_24_ = 0.36, *p* = 0.72; Figure 3E, left, one-sample *t*-test, *t*_15_ = 0.57, *p* = 0.58; no aging effect, unpaired *t*-test, *t*_39_ = 0.45, *p* = 0.66). Novelty after the weak encoding led to good LTM in the young rats (Figure 3D, right, one-sample *t*-test vs. chance, *t*_32_ = 5.17, *p* < 0.0001; paired *t*-test between no novelty and novelty, *t*_24_ = 5.19, *p* < 0.0001). However, novelty did not improve LTM in the middle-aged rats (Figure 3E, right, one-sample *t*-test with a chance level, *t*_15_ = 0.87, *p* = 0.4; paired *t*-test between no novelty and novelty, *t*_15_ = 0.2, *p* = 0.85), and a significant aging effect was observed (unpaired *t*-test, *t*_39_ = 2.06, *p* = 0.046). These results, collected through between-subject experiments, are consistent with our previous observation in a within-subject, longitudinal study (Gros and Wang, 2018).

### Reduced Neuronal Activation by Encoding and Modulating in Cognitive Aging

**FIGURE 4:**
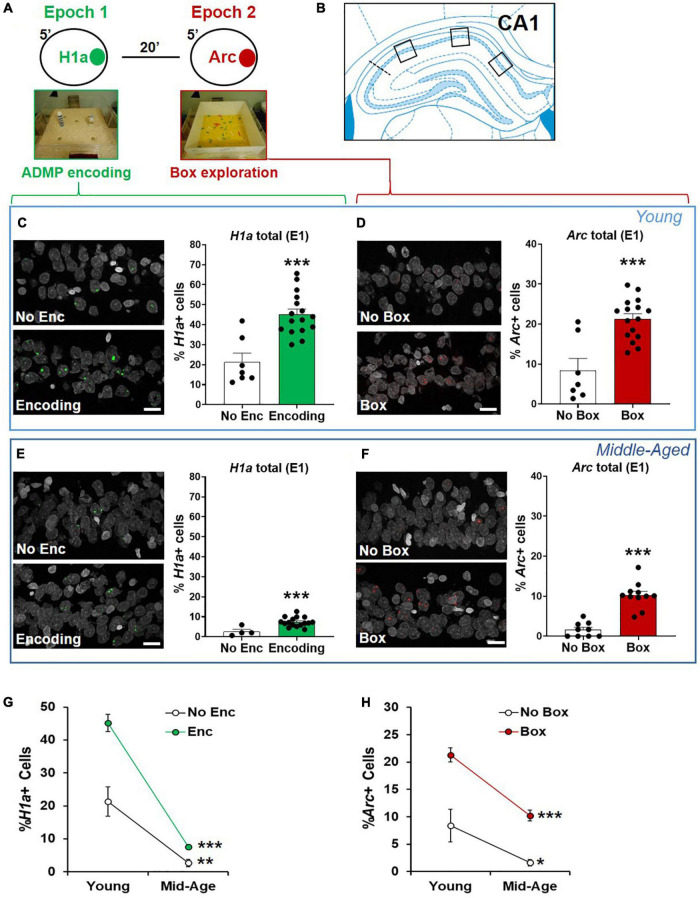
The proportions of CA1 neurons activated by ADMP encoding and novelty are reduced in aging. **(A)** Rats could be exposed to ADMP encoding (3 pellets) during epoch 1 for 5 min and then to exploration of a novel box during epoch 2 for 5 min. The rats were randomly assigned to the following groups: home cage (HC) control, encoding in epoch 1, novelty in epoch 2, encoding followed by exploration in a novel box, or encoding followed by exploration in a familiar box. Neurons activated by epoch 1 were referred to as *H1a* +, while neurons activated by epoch 2 were referred to as *Arc*+. **(B)** Images were collected in 3 areas of CA1. **(C)** Example images and the percentage of *H1a* + CA1 neurons in young rats. A scale bar: 10 μm. The young rats exposed to ADMP encoding (Enc) showed a higher percentage of *H1a* + neurons than the rats that did not receive ADMP encoding (No Enc, the HC rats, and the rats exposed only to novel box exploration). **(D)** Example images and the percentage of *Arc*+ CA1 neurons in the young rats. A scale bar: 10 μm. The young rats exploring a novel or familiar box (box) showed a higher percentage of *Arc*+ neurons than the rats that did not (no box, the HC rats, and the rats exposed only to ADMP encoding). **(E)** Example images and the percentage of *H1a* + CA1 neurons in the middle-aged rats. A scale bar: 10 μm. The middle-aged rats exposed to ADMP encoding (Enc) showed a higher percentage of *H1a* + neurons than the rats that did not receive encoding (No Enc, the HC rats, and the rats exposed only to novel box exploration). **(F)** Example images and the percentage of *Arc*+ CA1 neurons in the middle-aged rats. A scale bar: 10 μm. The middle-aged rats exploring a box showed a higher percentage of *Arc*+ neurons than the rats that did not (no box). **(G)** The percentage of *H1a* + CA1 neurons decreased with aging in control condition (No Enc, the HC rats) or after ADMP encoding (Enc). **(H)** The percentage of *Arc*+ CA1 neurons decreased with aging in control condition (no box, the HC rats, and the rats exposed only to ADMP encoding) or after exploration in a box (box). All data are presented as mean ± SEM. *p < 0.05, **p < 0.01, and ***p < 0.005.

Neuronal assemblies activated by memory encoding in epoch 1 and by novelty in epoch 2 were identified in the second experiment. In CA1 (Figure 4), strong encoding in the young rats increased the percentage of total *H1a*+ neurons (Figure 4C, No Enc = the rats in HC and the rats exposed to the box only, unpaired *t*-test, *t*_21_ = 4.85, *p* < 0.0001), and exploration in a box increased the percentage of total *Arc*+ neurons (Figure 4D, No Box = the rats in HC and the rats exposed to the encoding only, unpaired *t*-test, *t*_21_ = 4.7, *p* = 0.0001). The same pattern was seen in the middle-aged rats (Figures 4E,F; *H1a*: unpaired *t*-test, *t*_18_ = 3.61, *p* = 0.002; *Arc*: unpaired *t*-test, *t*_18_ = 7.1, *p* < 0.0001). However, the overall proportion of activated neurons by encoding or box exploration was much reduced by aging (Figures 4G,H, two-way ANOVA, age effect, H1a, *F*_1,39_ = 93.12, *p* < 0.0001; *Arc*, *F*_1,39_ = 33.93, *p* < 0.0001). This reduction was not due to an age-dependent change in movement as similar distance of walking was seen in the novel box in the young rats (1,375 ± 65.1 cm) and in the middle-aged rats (1,457 ± 135.3 cm; unpaired *t*-test, *t*_10_ = 0.55, *p* = 0.6).

The interaction between aging and the encoding effect was significant in the percentage of *H1a*+ neurons (Figure 4G, two-way ANOVA, interaction *F*_1,39_ = 10.72, *p* = 0.002), suggesting that aging reduces the baseline and encoding-triggered IEG expression, as well as the degree of encoding-triggered increase of IEG+ cells from the baseline. On the other hand, the interaction between aging and the box effect was not significant in the percentage of *Arc*+ neurons (Figure 4H, two-way ANOVA, interaction *F*_1,39_ = 1.9, *p* = 0.17), indicating that aging does not affect the degree of box exploration-triggered increase of IEG+ cells from the baseline. This would suggest that reduced basal activation of cells is seen in early aging, and, additionally, encoding-triggered cellular activation is reduced in early aging.

### Reduced Overlapping Population in Early Aging, an Effect Preferentially Correlated With the Encoding Population

**FIGURE 5:**
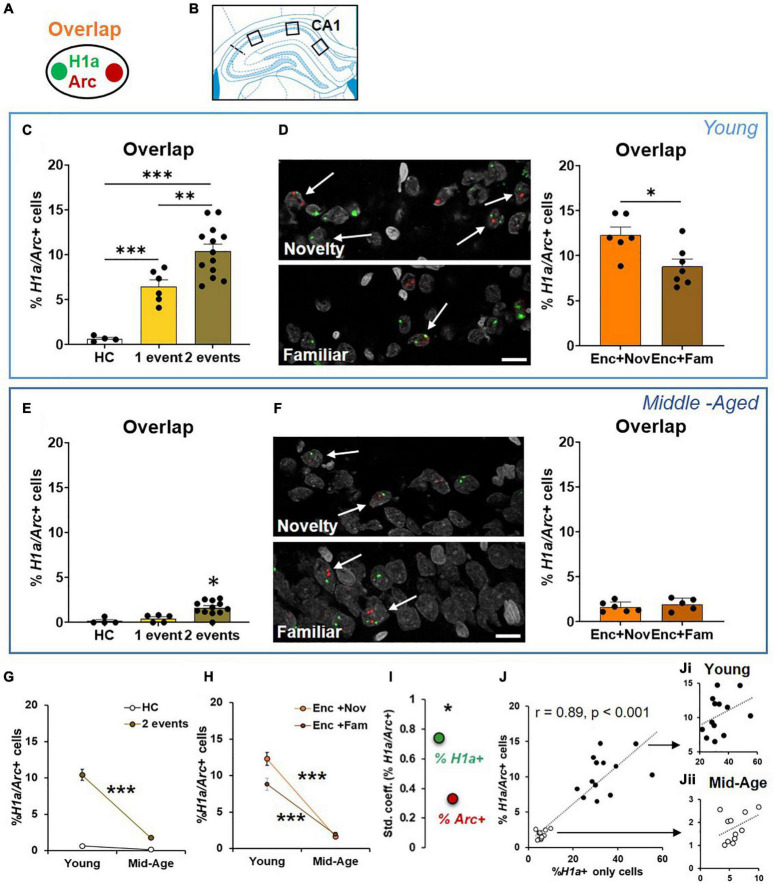
The cellular signature underpinning behavioral tagging is altered in aging. **(A)** Neurons activated by epoch 1 and epoch 2 were referred to as *H1a/Arc*+. **(B)** Images were collected in 3 areas of CA1. **(C)** In the young rats, the percentage of *H1a*/*Arc*+ CA1 neurons was highest in the rats receiving 2 events (Enc + Nov and Enc + Fam, 1 event = the rats exposed only to ADMP encoding or to novel box exploration). **(D)** An example and percentage of *H1a*/*Arc*+ CA1 neurons after novelty or familiarity exploration in the young rats. A scale bar: 10 μm. The percentage of neurons activated in the Enc + Nov group was higher than in the Enc + Fam group. **(E)** In the middle-aged rats, the percentage of *H1a*/*Arc*+ CA1 neurons was highest in the rats receiving 2 events (Enc + Nov and Enc + Fam, 1 event = the rats exposed only to ADMP encoding). **(F)** Example images and percentage of *H1a*/*Arc*+ CA1 neurons with novelty or familiarity exploration after ADMP encoding in the middle-aged rats. A scale bar: 10 μm. No difference was observed between the rats exposed to a novel or a familiar box. **(G)** The percentage of *H1a/Arc*+ CA1 neurons decreased with aging after 2 events exposure. **(H)** The percentage of *H1a*/*Arc*+ CA1 neurons in two sub-groups decreased with aging. **(I)** The standardized coefficients between the percentages of *H1a*/*Arc*+ and *H1a* + neurons (0.74) were significantly larger than which between the percentage of *H1a*/*Arc*+ and *Arc*+ neurons (0.33). **(J)** Correlation between the *H1a* + only neuronal population activated by ADMP encoding and the overlapping *H1a/Arc*+ population (black: young, white: middle-aged). **(Ji)** Correlation in the young rats. **(Jii)** Correlation in the middle-aged rats. Data are presented as mean ± SEM. *p < 0.05, **p < 0.01, and ***p < 0.005.

We next quantified neurons that co-expressed *H1a* and *Arc* in CA1 (Figures 5A,B). The young animals receiving events in both epochs had a higher percentage of co-expressing neurons than the animals receiving one event only, which was also higher than in the animals that remained in their home cage during both epochs (Figure 5C, one-way ANOVA *F*_2,20_ = 27.93, *p* < 0.0001; Tukey test: HC vs. 1 event, *p* = 0.003; HC vs. 2 events, *p* < 0.0001; 1 event vs. 2 events, *p* = 0.007). These results indicate that these two events engage an overlapping population of CA1 neurons.

We then asked if an effective memory-modulating event compared to an ineffective memory-modulating event would recruit a higher proportion of neurons that are co-activated by encoding. We observed that the group that explored a novel box after encoding showed a significantly higher percentage of co-expressing neurons than the group that explored a familiar box (Figure 5D, unpaired *t*-test, *t*_11_ = 2.86, *p* = 0.015). These results suggest that behavioral tagging engages an increased population of CA1 neurons co-activated by memory-encoding and memory-modulating events.

The middle-aged animals receiving two events in both epochs had a higher percentage of co-expressing neurons than the animals receiving one event only or remaining in their home cage (Figure 5E, Kruskal-Wallis H test H_3,20_ = 14.69, *p* < 0.0001; Dunn test: HC vs. 1 event, *p* > 0.99; HC vs. 2 events, *p* = 0.003; 1 vs. 2 events, *p* = 0.014). However, there was no group difference between novelty and familiarity (Figure 5F, unpaired *t*-test, *t*_9_ = 0.8, *p* = 0.44). To quantify the aging effect, we compared the data across age cohorts and found that the overall level of neurons co-expressing *H1a* and *Arc* in CA1 was reduced with aging (Figure 5G, two-way ANOVA *F*_1,28_ = 36.21, *p* < 0.0001). Both aging effect (young > middle-aged, two-way ANOVA, *F*_1,28_ = 36.21, *p* < 0.0001) and event effect (2 events > home cage, two-way ANOVA, *F*_1,28_ = 56.21, *p* < 0.0001) were significant. The interaction between the aging and event effects was also significant (Figure 5G, two-way ANOVA, *F*_1,28_ = 28.78, *p* < 0.0001), suggesting that aging reduces the degree of memory encoding- and memory modulation-triggered increase of overlapping IEG+ cells from the baseline.

Further focusing on behavioral tagging, “Enc + Nov” and “Enc + Fam” groups across age cohorts were compared (Figure 5H). The results showed a significant aging effect (two-way ANOVA, *F*_1,20_ = 160.9, *p* < 0.0001), a significant event effect (*F*_1,20_ = 5.32, *p* = 0.032) and a significant aging-event interaction (*F*_1,20_ = 7.54, *p* = 0.012). This indicates that the differential proportion of overlapping cells in the “Enc + Nov” and “Enc + Fam” groups is significantly reduced in aging. These results suggest that the behavioral tagging deficit observed in the middle-aged animals (Figure 3G) is associated with reduction in co-activated population of CA1 neurons.

To understand if the overlapping cells are correlated with H1a + only cells or Arc + only cells, Pearson correlations were performed. Both correlations were very high and significant (*H1a*+ only and *H1a/Arc*+, *r* = 0.89, *p* < 0.0001; *Arc*+ only and *H1a/Arc*+, *r* = 0.48, *p* < 0.05). To decipher if the contributions from *H1a*+ or from *Arc*+ were equal, regression analysis was performed (*r* = 0.87, *p* < 0.001). The standardized coefficients for *H1a/Arc*+ and *H1a*+ and for *H1a/Arc*+ and *Arc*+ were 0.74 and 0.33, respectively. A further test revealed that the coefficients with *H1a*+ were significantly higher than the coefficient with *Arc*+ (*p* < 0.05, Figure 5I), indicating that the proportion of *H1a*+ cells, compared to proportion of *Arc*+ cells, quantitatively contributes more to the overlapping cell population. This result indicates that the encoding-related neuronal population, compared to the modulation-related neuronal population, can explain more about the variation in sizes of the overlapping population.

A highly significant correlation between *H1a*+ only and overlapping populations was observed across 2 age groups (Figure 5J, *r* = 0.89, *p* < 0.001). To understand if this is driven by age difference or an effect that is already present in each age group, correlation was performed in individual groups and found to be insignificant (Figure 5Ji, young: *r* = 0.36; Figure 5Jii, middle-aged: *r* = 0.4; both *p* > 0.22). These results suggest that the size of encoding-related neuronal population explains about 80% of variation in the size of overlapping population from young to early aging, while only about 15% of the variation can be explained at each age point.

### Proportionally More Neurons in Distal CA1, Compared to Proximal CA1, Represent Both Memory Encoding and Effective Modulating Events in Young Rats

**FIGURE 6:**
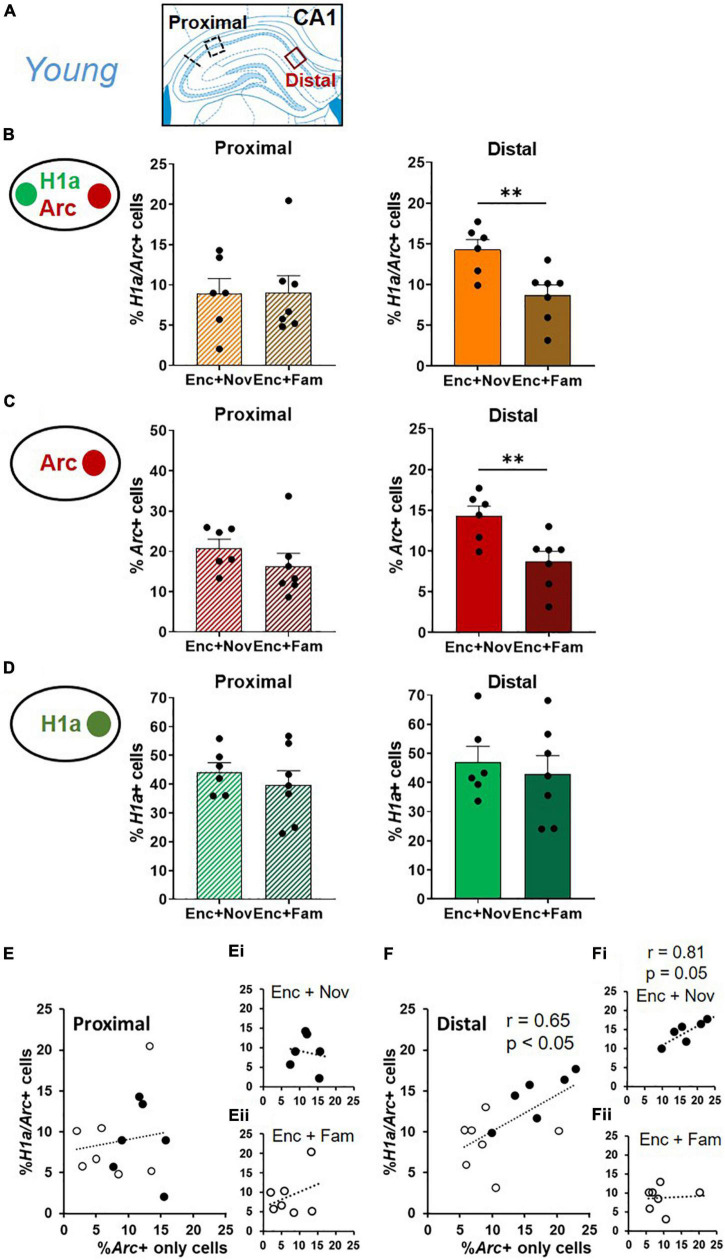
Distal CA1 activation during behavioral tagging in young rats. **(A)** Areas of CA1 where images were taken and separated as proximal and distal CA1. **(B)** The percentage of *H1a*/*Arc*+ neurons activated in the Enc + Nov group was higher in distal CA1. No difference was observed in proximal CA1. **(C)** The percentage of *Arc*+ neurons activated by box exploration was higher with novelty (Nov) than with familiarity (Fam) in distal CA1. No difference was observed in proximal CA1. **(D)** The percentage of *H1a* + neurons activated by ADMP encoding was similar between Enc + Nov and Enc + Fam groups in proximal and distal CA1. **(E)** Correlation between the *Arc*+ only neuronal population activated by box exploration and the overlapping population in proximal CA1. In white: the young rats exposed to familiarity exploration. In black: the young rats exposed to novelty exploration. **(Ei)** Correlation in the young rats exposed to novelty exploration. **(Eii)** Correlation in the young rats exposed to familiarity exploration. **(F)** Correlation between the *Arc*+ only neuronal population activated by box exploration and the overlapping population in distal CA1. In white: the young rats exposed to familiarity exploration. In black: the young rats exposed to novelty exploration. **(Fi)** Correlation in the young rats exposed to novelty exploration. **(Fii)** Correlation in the young rats exposed to familiarity exploration. Data are presented as mean ± SEM. ^**^p < 0.01.

To localize the source that contributed to the neuronal assemblies co-expressing *H1a* and *Arc* in CA1, distal and proximal areas of CA1 were compared (Figure 6A). Due to a significant reduction of percentages of IEG+ neurons in middle-aged animals (Figures 4, 5), this and subsequent analyses were only done in young animals to understand the circuit (CA1, CA3, and DG) and micro-circuit (distal vs. proximal areas of each region) mechanisms.

In the distal CA1, the proportion of overlapping neurons was higher in the “Enc + Nov” group than in the “Enc + Fam” group (Figure 6B, right, unpaired *t*-test, *t*_11_ = 3.2, *p* = 0.008). In the proximal CA1, this difference was not significant (Figure 6B, left, Mann-Whitney *U* test, U_11_ = 20, *p* = 0.95). This pattern was also seen in the percentage of *Arc*+ only neurons, with a higher percentage of *Arc*+ neurons in the distal CA1 in the “Enc + Nov” group (Figure 6C, Distal: unpaired *t*-test, *t*_11_ = 3.37, *p* = 0.006; Proximal: unpaired *t*-test, *t*_11_ = 1.14, *p* = 0.28). There was no group difference in either distal or proximal CA1 for the proportion of *H1a*+ only neurons (Figure 6D, Distal: unpaired *t*-test, *t*_11_ = 0.49, *p* = 0.64; Proximal: unpaired *t*-test, *t*_11_ = 0.74, *p* = 0.48). As the area difference was seen in overlapping and *Arc*+ only neurons, we ask if the size of the overlapping population is correlated with the size of *Arc*+ only population. Correlation was not significant in proximal CA1 (Figure 6E, *r* = 0.14, *p* = 0.65), but was significant in distal CA1 (Figure 6F, *r* = 0.65, *p* = 0.015). Moreover, a significant difference was observed between these two correlations (*z* = 2.12, *p* < 0.05). Sub-group analyses in these 2 regions revealed a marginally significant correlation in the Enc + Nov group in distal CA1 (Figure 6Fi, *r* = 0.81, *p* = 0.05), but not in proximal CA1 or in the Enc + Fam group (Figures 6Ei,Eii,Fii, all *r* < 0.33, *p* > 0.46). Together, these suggest that about 40% of the variation in the size of neuronal population representing two memory events can be explained by the neuronal population representing the modulating event in distal CA1, where the effect of behavioral tagging is more prominent.

### Novelty Potentiates the Signal Size in Nuclei Involved in Appetitive Delayed-Matching-to-Place Encoding but Does Not Change the Double-Labeled Population Among the Encoding Population

**FIGURE 7:**
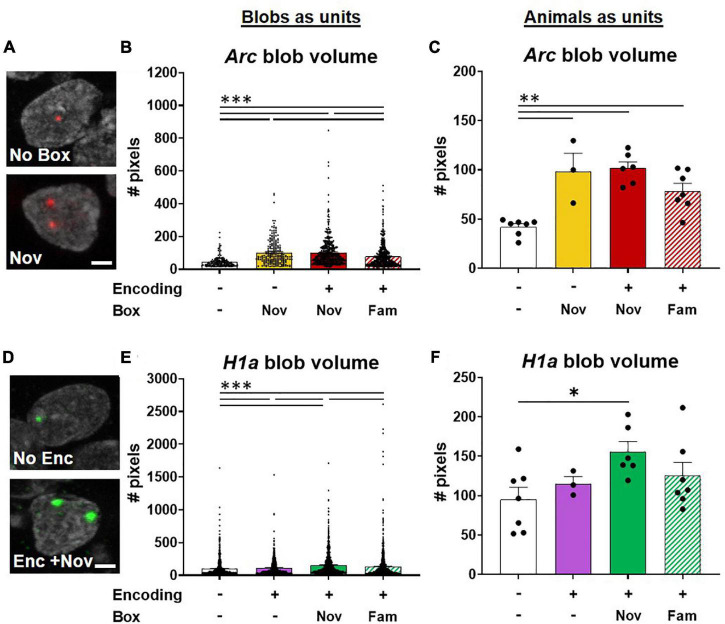
Potentiation of neuronal activations involved in ADMP encoding by novel box exploration. **(A)** Example images of *Arc* blob in neuron foci. A scale bar: 2 μm. **(B)** The volume of *Arc* signal in foci was higher with novelty exploration. **(C)** When animals, instead of blobs in neurons, were used as the analysis unit, the overall difference remained significant. The paired difference was significant in no box vs. other groups. **(D)** Example images of *H1a* blob in neuron foci. A scale bar: 2 μm. **(E)** Novel box exploration after encoding significantly more increased the volume of *H1a* signal than the ADMP encoding only. **(F)** When animals, instead of blobs in neurons, were used as the analysis unit, the overall difference became marginally significant. Only the difference between the No Enc and Enc + Nov groups remains significant. All data are presented as mean ± SEM. *p < 0.05, ^**^p < 0.01, and ^***^p < 0.005.

The volume of *H1a* and the *Arc* mRNA signal in foci have previously shown to reflect synaptic maturation (Montes-Rodríguez et al., 2013) and the relative firing rates within a population of neurons in response to a behavioral event (Miyashita et al., 2009; Witharana et al., 2018). To visualize if the memory events in epoch 1 or in epoch 2 would increase the IEG expression, the fluorescent signals in the nuclei were quantified (Figure 7).

The results of blobs as the analysis units demonstrated that the size of *Arc* blobs was largest with novelty than with familiarity and the home cage groups (Figures 7A,B, Kruskal-Wallis’s test, *F*_4,1256_ = 138.7, *p* < 0.0001, Dunn test *p* < 0.0001). However, no difference was observed when encoding was introduced before novelty exploration (Dunn test, Nov*vs* Enc + Nov, *p* > 0.99), indicating that prior ADMP encoding did not potentiate future IEG expression in the nuclei of activated neurons. When data were averaged for each animal, the overall effect remained highly significant (Figure 7C, one-way ANOVA, *F*_3,19_ = 14.01, *p* < 0.0001) and the animals with box exploration showed larger blob volumes than the animals without exploration (Figure 7C, Tukey tests *p* < 0.01).

The size of *H1a* blobs showed gradual changes depending on the number and type of memory events. The “Enc + Nov” group showed larger *H1a* blobs than the “Enc + Fam,” encoding alone, or no encoding groups (Figures 7D,E, Kruskal -Wallis’s test, H_4,3249_ = 106.5, *p* < 0.0001; Dunn test *p* < 0.001). When data were averaged for each animal, the overall effect became marginally significant (Figure 7F, one-way ANOVA, *F*_3,19_ = 2.78, *p* = 0.069), and only the animals that received “Enc + Nov” showed larger blob volumes than the animals without encoding (Tukey test, p = 0.045). Together, these results indicate that an effective memory modulating event (*i.e.*, novelty) increases the number of activated neurons (Figure 5D) and potentiates the IEG expression in the nuclei of the activated neurons (Figure 7).

We predicted that a significant overlapping population between encoding and novelty represents the cellular signature that contributes to memory persistence. To explore if this also occurs through novelty changing the proportion of *H1a*+ cells for encoding, we examined whether the rats exposed to novelty after the encoding showed a greater percentage of *H1a*+ cells to express *Arc* when behavioral tagging and capture occur. We quantified our data and found that the percentage of *H1a/Arc*+ cells among all *H1a*+ cells in the Enc + Nov group was not significantly different from the same measurement in the Enc + Fam group (the young rats, unpaired *t*-test, *t*_11_ = 1.36, *p* = 0.20; middle-aged rats; Mann-Whitney U test, U_9_ = 11, *p* = 0.54). Hence, the current data do not support the view that 5-min novelty at 20 min after encoding alters the encoding population. However, it is worth noting that when exploring the size of IEG expression in the nuclei, we observed an increase in the *H1a* size in the group that received novelty after encoding, compared to the group that received familiarity after encoding when each “blob” of *H1a* was used as the analysis unit.

### Less Activation of Neurons in CA3 Than in CA1 in Young Rats

**FIGURE 8:**
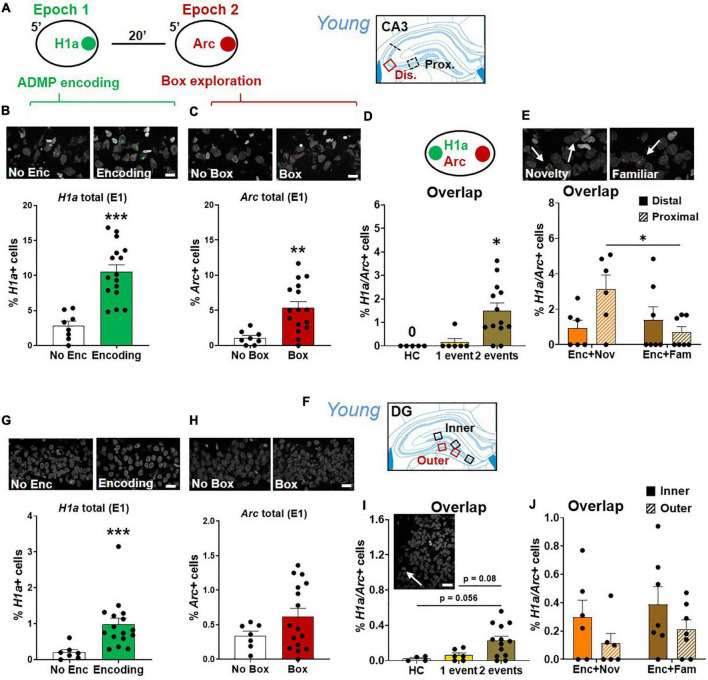
Proximal CA3 and DG activation during behavioral tagging in young rats. **(A)** The rats could be exposed to ADMP encoding (3 pellets) during epoch 1 for 5 min and then to exploration of a novel box during epoch 2 for 5 min. Images were collected in two areas of CA3. **(B)** Percentage of *H1a* + neurons. A scale bar: 10 μm. The rats exposed to ADMP encoding showed a higher percentage of *H1a* + neurons than the rats that did not receive encoding (No Enc, the HC rats, and the rats exposed only to novel box exploration) **(C)** Percentage of *Arc*+ neurons. A scale bar: 10 μm. The rats that explored a box showed a higher percentage of *Arc*+ neurons than the rats that did not (no box, the HC rats, and the rats exposed to ADMP encoding only). **(D)** The percentage of *H1a*/*Arc*+ neurons was highest in the rats receiving two events (Enc + Nov and Enc + Fam, 1 event = the rats exposed only to ADMP encoding or novel box exploration). **(E)** Percentage of *H1a/Arc*+ neurons between distal and proximal CA3. A scale bar: 10 μm. The percentage of *H1a*/*Arc*+ neurons was higher in the Enc + Nov group than in the Enc + Fam group in the proximal CA3. **(F)** Images were collected in five areas of DG. **(G)** Percentage of *H1a* + neurons. A scale bar: 20 μm. The rats exposed to ADMP encoding showed a higher percentage of *H1a* + neurons than the rats that did not receive encoding (No Enc, the HC rats, and the rats exposed only to novel box exploration). **(H)** Percentage of *Arc*+ neurons. A scale bar: 20 μm. Box exploration did not increase the percentage of *Arc*+ neurons than the rats that did not (no box, the HC rats, and the rats exposed only to ADMP encoding). **(I)** An example of *H1a*/*Arc*+ DG neurons. A scale bar: 20 μm. The percentage of *H1a*/*Arc*+ neurons was highest in the rats receiving 2 events (Enc + Nov and Enc + Fam, 1 event = the rats exposed to only ADMP encoding or novel box exploration). **(J)** No difference between inner and outer DG in the percentage of *H1a*/*Arc*+ neurons activated in the Enc + Nov group. All data are presented as mean ± SEM. *p < 0.05, **p < 0.01, and ***p < 0.005.

Results in CA3 (Figure 8A) showed that encoding increased the percentage of *H1a*+ neurons (Figure 8B, unpaired *t*-test, *t*_22_ = 5.16, *p* < 0.0001), and box exploration increased the percentage of *Arc*+ neurons (Figure 8C, unpaired *t*-test, *t*_22_ = 3.38, *p* = 0.003). Encoding following box exploration increased the percentage of *H1a*/*Arc*+ neurons (Figure 8D, Kruskal-Wallis H test, H_3,24_ = 12, *p* = 0.003; Dunn test: HC vs. 1 event, *p* > 0.99; HC vs. 2 events, *p* = 0.01; 1 event vs. 2 events, *p* = 0.027). However, the proportion of active neurons was much lower in CA3 than in CA1 (two-way ANOVA, *H1a*+, F_1,37_ = 14.69, *p* < 0.0001; *Arc*+, F_1,37_ = 124.97, *p* < 0.0001; *H1a/Arc*+, *F*_1,37_ = 191.3, *p* < 0.0001). Further focusing on the neuronal assemblies co-expressing *H1a* and *Arc* in distal and proximal areas of CA3, we showed that the group difference (encoding followed by novelty or familiarity) in the overlapping population was significant in the proximal CA3 but not in the distal CA3 (Figure 8E, two-way ANOVA, interaction effect, *F*_1,22_ = 5.58, *p* = 0.028; Tukey test, distal vs. proximal in the Enc + Nov group, *p* = 0.048). However, this group difference was insignificant in overall CA3 (unpaired *t*-test, *t*_11_ = 1.8, *p* = 0.1). These results indicate that encoding followed by an effective modulating event recruits more neurons in representing two events in the proximal CA3. To understand if this is related to the effect observed in distal CA1, a correlation analysis showed that the variance of overlapping population in proximal CA3 could significantly explain 52% of the variance in distal CA1 (*r* = 0.72, *p* < 0.01). This may suggest a functional circuit between proximal CA3 and distal CA1 for neurons co-representing the two memory events.

### Much Less Activation of Neurons in Dentate Gyrus Than in CA3 and CA1 in Young Rats

Results in DG (Figure 8F) showed that encoding increased the percentage of *H1a*+ neurons (Figure 8G, Mann-Whitney U test, U_21_ = 4, *p* < 0.001) and box exploration did not increase the percentage of *Arc*+ neurons (Figure 8H, unpaired *t*-test, *t*_21_ = 1.52, *p* = 0.15). Encoding and box exploration increased the percentage of *H1a*/*Arc*+ neurons (Figure 8I, one-way ANOVA, *F*_2,20_ = 4.46, *p* = 0.025; Tukey test: HC vs. 1 event, *p* = 0.9; HC vs. 2 events, *p* = 0.056; 1 event vs. 2 events, *p* = 0.08). Ramirez-Amaya et al. (2013) showed a prolonged Arc expression in DG granule cell nuclei after exposure in a novel box at 30 min before euthanasia (i.e., epoch 1). This can lead to about 0.7% of double cells in granule cells. In our study, we found 0.06% of overlapping cells in granule cells in the group that received encoding in epoch 1. The difference across studies is likely due to our epoch 1 being in a familiar arena where the animals had frequent exposure. When factoring in double cells caused by epoch 1 in the analysis of Figure 8I, the conclusion that 2 events trigger more overlapping cells than 1 event does remain unchanged.

The proportion of active neurons was much lower in DG than in CA3 (two-way ANOVA, *H1a*+, F_1,37_ = 73.1, *p* < 0.0001; *Arc*+, F_1,37_ = 21.42, *p* < 0.0001; *H1a/Arc*+, F_1,37_ = 9.03, *p* = 0.005), and further lower than in CA1 (two-way ANOVA, *H1a*+, F_1,36_ = 278.22, *p* < 0.0001; *Arc*+, F_1,36_ = 296.61, *p* < 0.0001; *H1a/Arc*+, F_1,36_ = 257.79, *p* < 0.0001). Furthermore, focusing on the neuronal assemblies co-expressing *H1a* and *Arc* in the inner layer or the outer layer of DG granular neurons (Figure 8J), there was no significant group difference in overlapping population, no significant layer difference, or interaction (two-way ANOVA, all F < 3.25, *p* > 0.09). Moreover, no group difference was observed in total DG (unpaired *t*-test, *t*_11_ = 1.04, *p* = 0.32). Correlation analyses of the overlapping population between inner or outer DG and proximal CA3 or between inner or outer DG and distal CA1 did not reveal any significance.

**FIGURE 9:**
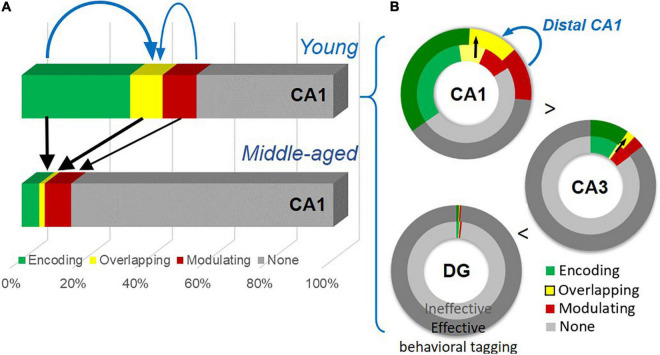
A summary of the age-dependent neuronal changes and the gradient of neuronal engagement in subregions of the hippocampus. **(A)** Neuronal activation by the encoding, by both events (thick black arrows), and by the modulating event (a thin black arrow) shows significant reduction with age. The encoding population (a thick blue arrow), compared to the modulating population (a thin blue arrow), is preferentially more correlated with the overlapping population. **(B)** Effective behavioral tagging in young animals leads to more overlapping population (black arrows) in CA1 and CA3. There is a gradient of active cell populations from DG to CA1. The modulating population (a blue arrow) is correlated with the overlapping population in distal CA1.

To visualize age-dependent change in the size of active neuronal assemblies, we summarized the percentage of encoding, overlapping, and modulating populations in CA1 from combined “Enc + Nov” and “Enc + Fam” groups (Figure 9A). To highlight the gradient across hippocampal regions, we summarized these percentages in concentric rings reflecting effective (“Enc + Nov”) and ineffective (“Enc + Fam”) behavioral tagging (Figure 9B). The size of active neuronal populations increases from DG to CA3 to CA1 (one-way ANOVA, Enc + Nov, F_2,15_ = 171.4, *p* < 0.0001; Enc + Fam, F_2,18_ = 135.9, *p* < 0.0001).

## Discussion

Age-dependent cognitive decline can manifest in different aspects of memory processing and with neuronal changes at the circuit level. Our study shows a decline of memory persistence in early aging in an appetitive spatial paradigm and impairment in behavioral tagging (Figure 3). Key findings are summarized in Figure 9. At the cellular level (Figure 9A), an age-dependent significant reduction in neurons expressing IEGs is seen after memory encoding and modulating events, with the encoding-related (Figure 4G) and overlapping populations (Figure 5H) showing further changes from the basal change. The size of the encoding-activated population, more so than the modulation-activated population, can significantly account for the variation in the size of the overlapping population across two age groups (Figure 5I). At the circuit level (Figure 9B), progressively more neurons are activated in the tri-synaptic circuits in the hippocampus from DG to CA1. An effective modulating event followed by encoding leads to memory persistence (Figure 3D) and more overlapping neurons in CA1 and CA3 (Figures 5D, 8E) in the young animals. The engagement of overlapping neuronal population in distal CA1 is more prominent than in proximal CA1 (Figure 6B), which correlates with the size of the modulation-activated population (Figure 6F).

This study uses a cross-sectional design due to the non-recovery nature for neuronal labeling. Peri-encoding novelty was used to study if it facilitates memory persistence (Moncada and Viola, 2007; Wang et al., 2010). A novel experience is shown to activate transcription factors that are relevant to learning and memory (Kinney and Routtenberg, 1993), to increase dopamine release in the hippocampus (Ihalainen et al., 1999), and to trigger changes in plasticity-related proteins (Moncada and Viola, 2006; Redondo and Morris, 2011). It is hypothesized that memory encoding tags synapses, which then enable capturing of plasticity-related proteins triggered by the novel experience happening around encoding and lead to lasting memory (review in Wang and Morris, 2010; Okuda et al., 2020). Blocking dopamine D1/D5 receptors or inhibiting protein synthesis in the hippocampus can prevent the novelty from improving memory (Moncada and Viola, 2007; Wang et al., 2010). The finding on age-dependent decline in memory persistence and behavioral tagging replicates our previous study using a longitudinal design (Gros and Wang, 2018). While memory deficits have been well documented at a later stage of aging, our ADMP paradigm, similar to the everyday experience in humans, sensitively detects early cognitive aging.

A key cellular signature for behavioral tagging is hypothesized to involve overlap of neuronal populations activated by memory encoding and modulating events. Nomoto et al. (2016) showed that the overlapping population of CA1 cells recruited by novel object recognition and by novel context exploration increases when behavioral tagging occurs. Here, we demonstrate this effect with a behavioral task that simulates everyday spatial memory and show that the overlapping population of neurons recruited by the behavioral tagging paradigm is evident in CA1 and CA3. A greater population of cells that are both *H1a*+ and *Arc*+ is seen in the effective behavioral tagging group than in the ineffective behavioral tagging group in the young animals. When zooming in and comparing these groups in distal and proximal CA1, this pattern is apparent in the distal CA1. Moreover, there is a higher proportion of *Arc*+ cells seen in the effective behavioral tagging group than in the ineffective behavioral tagging group in distal CA1. The correlation between the percentage of double positive cells and the percentage of *Arc*+ cells is also significant. This suggests that the higher proportion of double positive cells is likely driven by higher proportion of cells activated by novelty, and these are the underlying cellular signatures for memory persistence to occur through behavioral tagging and capture.

Previous behavioral evidence points to an encoding process being affected in early aging (Gros and Wang, 2018). Cellular data presented here corroborate this hypothesis. Particularly, memory encoding-triggered neuronal assemblies and overlapping assemblies triggered by encoding and modulating events are further reduced in aging CA1. Decreased hippocampal response to excitatory stimulation has been reported in middle age (Gonzales et al., 1991; see review in Shetty and Sajikumar, 2017). It is conceivable that an age-dependent decrease in NMDA receptor-mediated response underlies the decreased assembly size in our study. As aging is known to change gene expression, it is possible that the reduced assembly is associated with these changes. The decrease in the basal condition could be related to a reduction of certain gene expression in extra-cellularly regulated signaling and in early response transcription factors in middle age (Blalock et al., 2003). The decrease in the encoding condition could be related to reduction of gene expression that is significantly correlated with spatial memory performance, such as early growth response 2 and RDJ1 in protein trafficking (Blalock et al., 2003). With progression in aging, it is possible that a decline in consolidation mechanisms takes place later on (Ward et al., 1999; Monti et al., 2005). Synaptic plasticity-induced gene changes (Ryan et al., 2015) and reduction in IEG expression have been seen in advanced aging at 22 months or older (Desjardins et al., 1997; Penner et al., 2011, 2016; Chawla et al., 2013).

The observed age-dependent reduction of IEG+ CA1 population is consistent with a previous study, quantifying the decreased level of *Arc* mRNA expression with aging (Penner et al., 2011). A few studies from the same group, however, do not find a significant reduction of IEG+ population in the CA1 (Penner et al., 2011, 2016; Marrone et al., 2012; Gheidi et al., 2020). Several factors can account for the difference. First, our approach aimed at detecting changes from early to middle adulthood. The young animals in our study were much younger than those in the previous studies (e.g., 8 months old) (Penner et al., 2011, 2016; Marrone et al., 2012; Hartzell et al., 2013; Gheidi et al., 2020). While we reported early cognitive aging in this study, further comparison between midlife and more advanced aging has not been done. Second, the animals were encouraged to explore the entire novel environment by manually placing them in every zone of the box in previous studies (Penner et al., 2011, 2016; Marrone et al., 2012; Gheidi et al., 2020). In the current study, they freely explored the environment and were more frequently handled and/or underwent more behavioral training in the ADMP task. Thus, the procedural novelty or stress due to the endpoint handling for tissue collection would be greatly attenuated, which could increase the sensitivity in detecting age-dependent changes. Behavioral experiments in this study were performed during the light phase based on previous behavioral tagging work (Wang et al., 2010; Salvetti et al., 2014; Nomoto et al., 2016; Takeuchi et al., 2016; Gros and Wang, 2018; Orlandi et al., 2020; Tintorelli et al., 2020). Total sleep time, rapid-eye-movement (REM) sleep duration, and non-REM duration remain unchanged at midlife in rats, although a subtle aspect of non-REM sleep might start to show difference (Mendelson and Bergmann, 1999). Future research can look into whether behavioral tagging and age-related cellular change occur in the dark cycle, although a recent study has suggested age-dependent change in the sleep pattern might be more apparent in the dark cycle (Soltani et al., 2019).

The size and the intensity of *H1a* foci signal detection by FISH reflect the amount of mRNA present in single neurons (Montes-Rodríguez et al., 2013) and that these can change with behavioral experience (Witharana et al., 2018). In our study, the exploration of a novel environment increases the proportion of *Arc*+ neurons, as well as larger *Arc*+ volumes in CA1. The increase of the volume of *H1a* signal induced by encoding with novelty could reflect an increase of the amount of mRNA produced by a single neuron. It is unusual that a novel event retrospectively changes the *H1a* expression induced by encoding. It could be that the mRNA produced as a result of the neuronal activation during encoding is immediately available and quickly expressed during novelty exploration to influence subsequent rapid expression of *H1a* mRNA. Future research can investigate whether brain-derived neurotrophic factor (BDNF) and cAMP response element-binding proteins (CREB) that are shown to influence *H1a* expression (Sato et al., 2001; Mahan et al., 2012; Serchov et al., 2016) are involved in this process. Bekinschtein et al. (2008) showed that synthesis of BDNF during the late protein synthesis-dependent phase is crucial for persistence of memory storage and indicated that BDNF is a key molecule involved in memory persistence. Our and other behavioral tagging paradigms investigate events around encoding that enable memory to persist for a long term and have demonstrated an earlier phase of protein-synthesis dependency (Moncada and Viola, 2007; Ballarini et al., 2009; Wang et al., 2010). We currently do not know if novelty-enhanced persistence of the appetitive spatial memory seen in the young animals also engages a late phase of protein-synthesis dependency, which will require future investigation.

The gradient of IEG+ neuronal assemblies in the hippocampal tri-synaptic circuit in our paradigm is similar to previous observations using box exploration (Vazdarjanova and Guzowski, 2004; Miyashita et al., 2009; Penner et al., 2011; Nalloor et al., 2012). More overlapping neurons in the CA1 than in CA3 and DG suggest sparse representation in the two latter regions. Evidence from both electrophysiological recording of granular cells and imaging of activity-dependent IEG expression shows sparse coding in DG during spatial exploration (Jung and McNaughton, 1993; Chawla et al., 2005; Diamantaki et al., 2016). DG activation in our study is fairly low and is closer to the range of one previous study when positional cues are used (e.g., <2% in Hoang et al., 2018) than other studies that show higher activation (e.g., 2 to 5% in Chawla et al., 2005, 2018; Gheidi et al., 2013; Penner et al., 2016). When looking at box exploration-induced *Arc* mRNA expression, the percentage of positive cells in this study is lower than earlier studies (Chawla et al., 2005, 2018; Ramírez-Amaya et al., 2005). The difference likely reflects the behavioral procedures that were used to induce IEG expression. Previous studies often encourage animals to explore the entire novel environment by manually placing the animals in every zone of the box (Chawla et al., 2005; Ramírez-Amaya et al., 2005; Marrone et al., 2012; Gheidi et al., 2013). In the current study, the animals voluntarily explored the box with novel substrates on the floor without being regularly placed by the experimenter. It is likely that difference in procedure-led neurotransmitter release (Ihalainen et al., 1999), stress level (Balcombe et al., 2004), and perception of the environmental cues (Hoang et al., 2018) could contribute to the different levels of cellular activation. The role of CA2 between CA1 and CA3 in behavioral and cellular tagging has not been studied here and warrants future investigation with genetic markers to delineate this region. CA2 has been shown to be sensitive to representing contextual changes (Wintzer et al., 2014), and the projection from the supramammillary nucleus of hypothalamus to CA2 is shown to detect social novelty of an environment (Chen et al., 2020).

A larger overlapping population has been seen in the distal CA1 and proximal CA3 than their counterparts after encoding followed by novelty. This is also prominent with novelty-triggered *Arc*+ neurons in the distal CA1, which is correlated with the overlapping population. This suggests that, at the circuit level, the memory-modulating event provides critical input for determining the size of neural assemblies in co-representing two memory events. Recruitment of the distal CA1-proximal CA3 network has been shown in a non-spatial recognition task (Nakamura et al., 2013). Distal CA1 and proximal CA3 preferentially process non-spatial information from lateral entorhinal cortex based on IEGs and electrophysiological studies (Burke et al., 2011; Ito and Schuman, 2012; Sauvage et al., 2013; Nakazawa et al., 2016). Exploration of novel objects leads to neuronal activation in distal CA1 and proximal CA3 (Ito and Schuman, 2012; Hoang et al., 2018). It is likely that novelty in our paradigm, which is induced by new substrates with unique textures on the floor, has been registered in the “what/object” hippocampal circuit. The findings further suggest: (1) correlation in neurons co-representing two memory events in inner DG and proximal CA3 contributes to correlated assembly sizes in proximal CA3 and distal CA1, (2) increased neurons representing novelty in distal CA1 contribute to increased neurons co-representing two memory events in the same area, and (3) these two processes work in concert to enable behavior tagging and achieve memory persistence.

To conclude, our findings support the notion that an overlapping neuronal population, preferentially in the distal CA1 and the proximal CA3, is associated with behavioral tagging in facilitating memory persistence in young animals (behavioral results in Figure 3D, consistent with findings from a similar paradigm in Wang et al., 2010; Gros and Wang, 2018, and with other behavioral observations, see reviews in Wang and Morris, 2010; Moncada et al., 2015; Okuda et al., 2020). Moreover, this cellular signature is altered in early aging, primarily through a downregulation of the memory encoding-activated cellular network. This paradigm opens the door for future investigation of the molecular mechanisms (Castello-Waldow et al., 2020; Cizeron et al., 2020) in incorporation of neurons into a memory trace to enable memory persistence and in improving cognitive aging.

## Data Availability Statement

The data supporting the conclusions of this article will be made available by contacting the corresponding author.

## Ethics Statement

The animal study was reviewed and approved by the Bioresearch and Veterinary Services, University of Edinburgh, and adhered to the UK Animals (Scientific Procedures) Act 1986, Amendment Regulations 2012.

## Author Contributions

AG and S-HW designed the experiments. AG, AL, VH, NW, and S-HW performed the research and analyzed the data. ME contributed to the analytic tools. TM contributed to the catFISH protocols and training. AG and S-HW wrote the article. TM, VH, and AL revised it. S-HW proposed and secured funding for this project. All authors contributed to the article and approved the submitted version.

## Conflict of Interest

The authors declare that the research was conducted in the absence of any commercial or financial relationships that could be construed as a potential conflict of interest.

## Publisher’s Note

All claims expressed in this article are solely those of the authors and do not necessarily represent those of their affiliated organizations, or those of the publisher, the editors and the reviewers. Any product that may be evaluated in this article, or claim that may be made by its manufacturer, is not guaranteed or endorsed by the publisher.

## Supplementary Material

Supplementary Figure 1 ADMP training does not change the proportion of *Arc*+ neurons after novel box exploration**. (A)** *Arc* mRNA expression in cytoplasm nuclei was used to quantify neuronal activation after novel box exploration during epoch 1 or epoch 2, respectively. **(B)** An example of *Arc*+ CA1 neurons (red). A scale bar: 10 μm. **(C)** ADMP training did not change the percentage of *Arc*+ neurons activated by novel box exploration during epoch 1. **(D)** ADMP training did not change the percentage of *Arc*+ neurons activated by novel box exploration during epoch 2.

Supplementary Figure 2 Evaluation of different methods for catfish analysis. **(A-D)** Manual detection of overlapping CA1 neurons after re-exposure to the same novel box. **(A)** Left. Percentage of neurons expressing *Arc* in cytoplasm (with and without co-expressing *Arc* in foci) showed increase by novelty during epoch 1. Right. Percentage of neurons expression *Arc* in foci (with and without co-expressing *Arc* in cytoplasm) showed increase by novelty during epoch 2. **(B)** Left. Percentage of neurons expressing *Arc* only in cytoplasm (without co-expressing *Arc* in foci) showed increase by novelty during epoch 1. Middle. Percentage of neurons expressing *Arc* only in foci (without co-expressing *Arc* in cytoplasm) showed increase by novelty during epoch 2. Right. Percentage of neurons co-expressing *Arc* in cytoplasm and foci showed an increase when novelty was introduced two times. **(C)** Left. Percentage of neurons expressing *H1a* (with or without co-expressing *Arc*) showed increase by the novel box exploration in epoch 1. #_1_, Box + HC vs. HC + Box, *p* = 0.054. Right. Percentage of neurons expressing *Arc* (with or without co-expressing *H1a*) showed increase by the novel box exploration in epoch 2. **(D)** Left. Percentage of neurons expressing *H1a* only (without co-expressing *Arc*) showed increase by novelty during epoch 1. #_2_, Box + HC vs. Box + Box, *p* = 0.075. Middle. Percentage of neurons expressing *Arc* only (without co-expressing *H1a*) showed increase by novelty during epoch 2. Right. Percentage of neurons co-expressing *H1a/Arc* showed an increase when novelty was introduced two times.
