# Supplementary figures and images for: Behavioral and Cellular Tagging in Young and in Early Cognitive Aging
Source: Front Aging Neurosci. 2022 Feb 24;14:809879. doi: 10.3389/fnagi.2022.809879 (PMC8907879; doi:10.3389/fnagi.2022.809879)

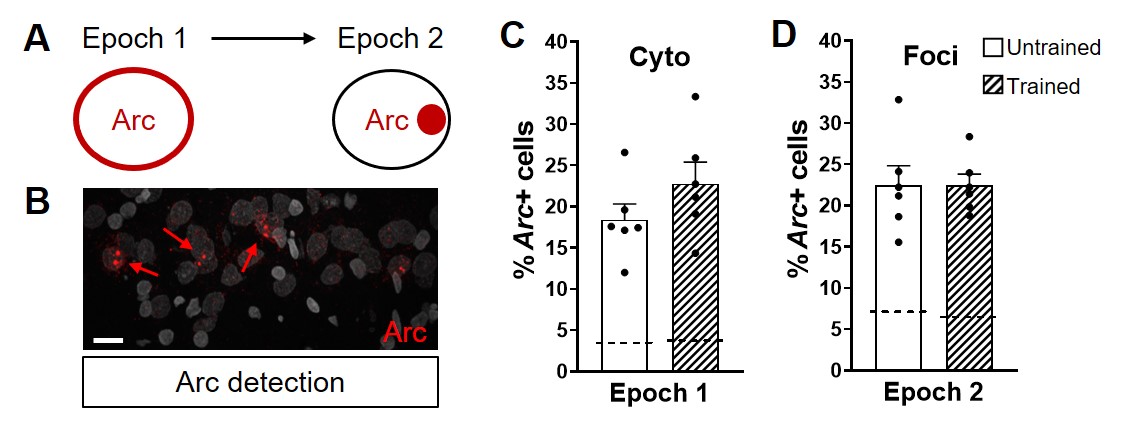

Supplement: Supplementary Figure 1 — ADMP training does not change the proportion of Arc+ neurons after novel box exploration. (A) Arc mRNA expression in cytoplasm nuclei was used to quantify neuronal activation after novel box exploration during epoch 1 or epoch 2, respectively. (B) An example of Arc+ CA1 neurons (red). A scale bar: 10 μm. (C) ADMP training did not change the percentage of Arc+ neurons activated by novel box exploration during epoch 1. (D) ADMP training did not change the percentage of Arc+ neurons activated by novel box exploration during epoch 2. [file Image_1.JPEG]

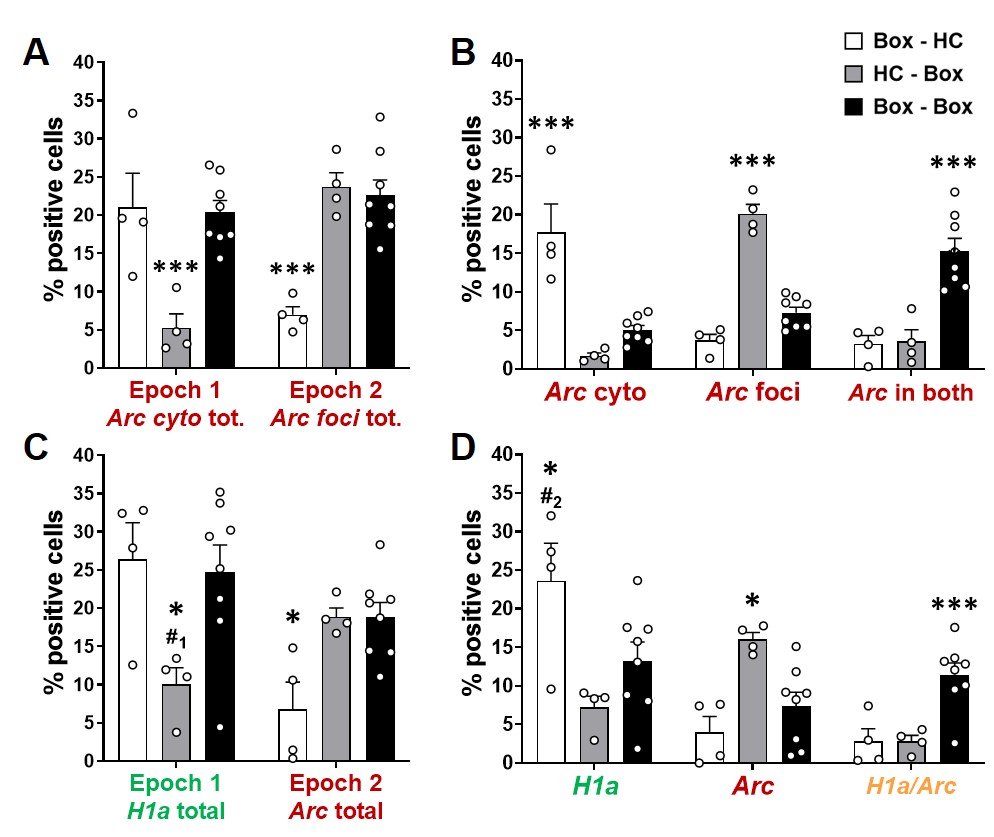

Supplement: Supplementary Figure 2 — Evaluation of different methods for catfish analysis. (A-D) Manual detection of overlapping CA1 neurons after re-exposure to the same novel box. (A) Left. Percentage of neurons expressing Arc in cytoplasm (with and without co-expressing Arc in foci) showed increase by novelty during epoch 1. Right. Percentage of neurons expression Arc in foci (with and without co-expressing Arc in cytoplasm) showed increase by novelty during epoch 2. (B) Left. Percentage of neurons expressing Arc only in cytoplasm (without co-expressing Arc in foci) showed increase by novelty during epoch 1. Middle. Percentage of neurons expressing Arc only in foci (without co-expressing Arc in cytoplasm) showed increase by novelty during epoch 2. Right. Percentage of neurons co-expressing Arc in cytoplasm and foci showed an increase when novelty was introduced two times. (C) Left. Percentage of neurons expressing H1a (with or without co-expressing Arc) showed increase by the novel box exploration in epoch 1. #1, Box + HC vs. HC + Box, p = 0.054. Right. Percentage of neurons expressing Arc (with or without co-expressing H1a) showed increase by the novel box exploration in epoch 2. (D) Left. Percentage of neurons expressing H1a only (without co-expressing Arc) showed increase by novelty during epoch 1. #2, Box + HC vs. Box + Box, p = 0.075. Middle. Percentage of neurons expressing Arc only (without co-expressing H1a) showed increase by novelty during epoch 2. Right. Percentage of neurons co-expressing H1a/Arc showed an increase when novelty was introduced two times. [file Image_2.JPEG]
